# Artificial Intelligence for Predicting Perioperative Outcomes in Cardiac Surgery: A Systematic Review

**DOI:** 10.1177/15569845261457046

**Published:** 2026-07-13

**Authors:** Jason Ha, Hunaid A. Vohra

**Affiliations:** 1 University of Bristol, United Kingdom

**Keywords:** artificial intelligence, machine learning, deep learning, cardiac surgery, cardiothoracic surgery

## Abstract

**Objective:**

Cardiac surgery carries a significant risk of complications and mortality. Artificial intelligence (AI), particularly machine learning (ML), is increasingly being explored to enhance perioperative risk prediction and support clinical decision-making. This systematic review evaluates the clinical applications, predictive performance, and limitations of AI models in cardiac surgery.

**Methods:**

PubMed and Embase were searched for studies published between January 2020 and July 2025. Of 939 records identified, 178 studies met the inclusion criteria following screening and full-text review. Included studies applied AI to predict clinical outcomes in patients undergoing cardiac surgery. Key outcomes assessed were model performance metrics and their clinical utility.

**Results:**

Among the 178 included studies, 114 (64%) were conducted in the United States or China. Most studies (*n* = 168, 94%) used retrospective designs and focused on adult populations. Random forest (*n* = 82, 46%), logistic regression (*n* = 82, 46%), and eXtreme Gradient Boosting (*n* = 70, 39%) were the most frequently used algorithms. AI applications primarily targeted the prediction of postoperative complications (*n* = 102, 57%) and mortality (*n* = 70, 39%), with common outcomes including acute kidney injury and stroke. ML models consistently outperformed traditional clinical risk scores (*n* = 39). SHapley Additive exPlanations was the most common interpretability method (*n* = 66, 37%). Only 26% of studies included external validation, and just 19% adhered to TRIPOD guidelines.

**Conclusions:**

AI models demonstrate superior predictive performance in cardiac surgery compared with traditional risk scores, but concerns regarding validation, transparency, and generalizability must be addressed to enable implementation.

## Graphical abstract

**Graphical Abstract:**
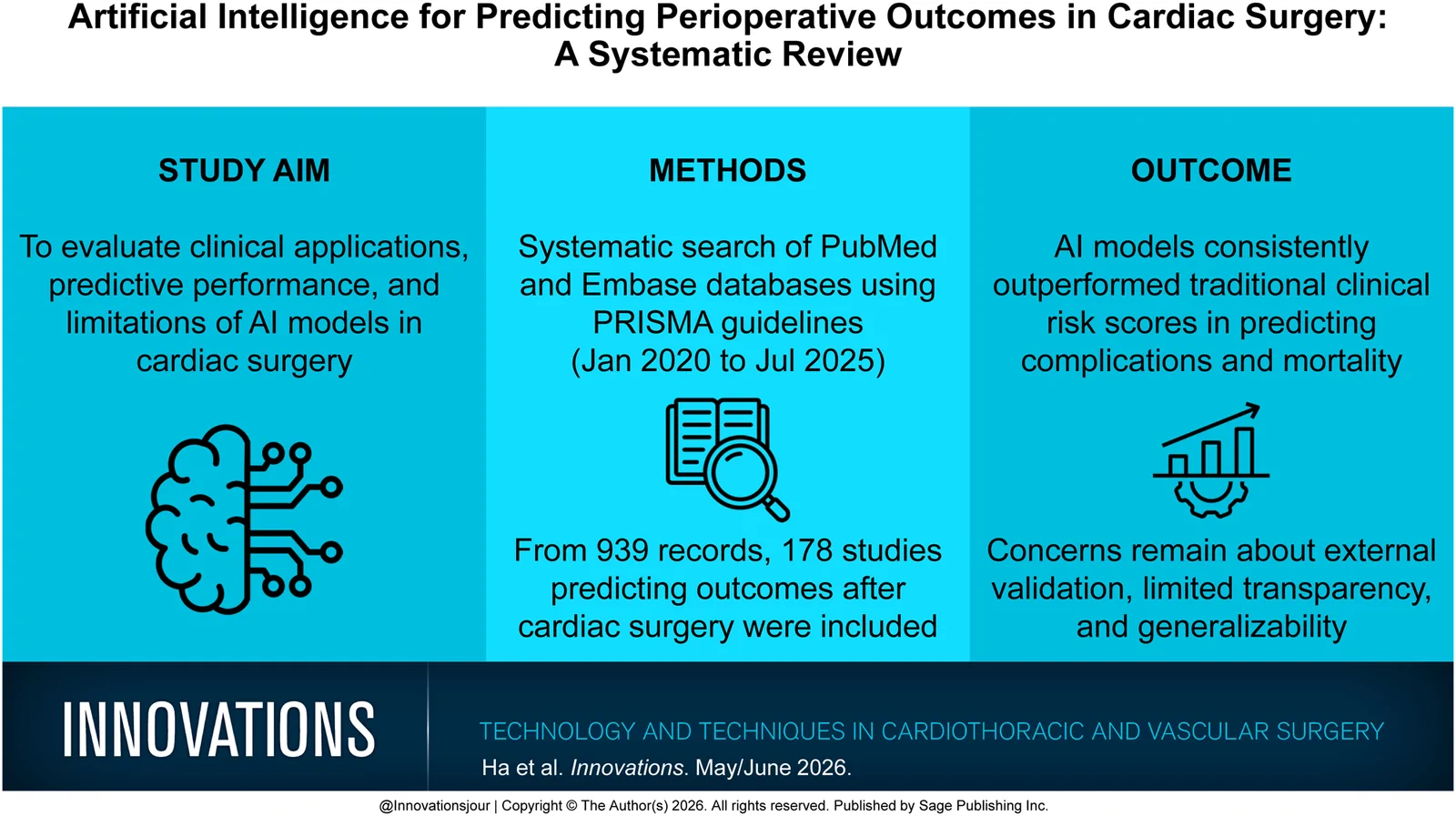
This is a visual representation of the abstract.

**Central Message**

AI models, especially tree-based ones, outperform traditional risk models in predicting cardiac surgery outcomes. Most studies were retrospective and single country, with limited external validation and poor TRIPOD adherence. Prospective, multicenter studies with better transparency and interpretability are needed for safe clinical use.

## Introduction

More than 1 million cardiac surgical procedures are performed globally each year, with the most common being coronary artery bypass grafting (CABG), followed by valvular heart surgery and congenital heart surgery.^1^ These surgeries often involve high-risk patients with multiple comorbidities, making accurate risk stratification, perioperative planning, and postoperative monitoring essential. However, traditional methods for risk stratification and perioperative management can be limited, prompting exploration of innovative solutions to enhance clinical decision-making and patient outcomes.^2^

Artificial intelligence (AI), encompassing machine learning (ML) and deep learning (DL), has emerged as a transformative tool in modern medicine. ML algorithms analyze data to identify patterns and make predictions, either by learning from labeled examples (supervised learning) or by detecting intrinsic structures within unlabeled datasets (unsupervised learning). DL, a subset of ML inspired by human neurons, employs multiple interconnected processing layers to capture complex, nonlinear relationships in datasets.^3^ Compared with traditional risk prediction methods, AI models have demonstrated superior accuracy and predictive performance across various medical disciplines. These computational techniques can uncover subtle patterns within patient data that traditional statistical methods might miss, providing surgeons with a more precise clinical picture.^4^

Despite these promising advancements, the role and effectiveness of AI applications within cardiac surgery require comprehensive evaluation. Robust assessment of AI models depends on rigorous validation, transparent reporting, and reproducibility. External validation across different populations and clinical settings is essential to assess generalizability beyond the development cohort. Adherence to reporting guidelines such as the Transparent Reporting of a multivariable prediction model for Individual Prognosis Or Diagnosis for AI (TRIPOD-AI) ensures transparency in model development, including details on data preprocessing, feature engineering, hyperparameter selection, and validation strategies.^5^ Furthermore, the availability of code, trained model weights, and clear documentation of the AI pipeline are critical for independent reproduction and benchmarking of published models. Therefore, this systematic review aims to assess the clinical applications and outcomes of AI in cardiac surgery while identifying current limitations and outlining directions for future research.

## Methods

This systematic review was conducted in accordance with the Preferred Reporting Items for Systematic Reviews and Meta-Analyses (PRISMA) guidelines. This review was registered on PROSPERO (CRD420251081627). The literature search was conducted in PubMed and Embase (via Ovid) up to July 2025, restricted to studies published from January 2020 onward to include only recent advancements. The search combined controlled vocabulary (MeSH and Emtree terms) and free-text keywords related to AI (e.g., “Artificial Intelligence,” “Machine Learning,” “Deep Learning,” “Computer Vision,” “Neural Networks, Computer,” “AI”) and cardiac surgery (e.g., “Cardiac Surgical Procedures,” “cardiac surgery,” “cardiothoracic surgery,” “heart surgery”). Boolean operators were used to combine terms. Additional papers were also found from the reference lists of the included papers.

Studies were included if they discussed the following:

1. Population: Human patients of any age (adults and pediatric) undergoing cardiac surgery, including open or minimally invasive procedures.
2. Intervention: Use of AI tools, including ML or DL, applied at any phase of the surgical process (preoperative, intraoperative, or postoperative).
3. Comparison: Traditional clinical risk models or standard care practices without AI assistance, if available.
4. Outcomes: Either predictive performance or clinical outcomes, including risk stratification, postoperative complications, or system-level outcomes such as cost and time efficiency.

Animal or bench-top studies, work that applied AI solely to nonsurgical cardiology tasks, papers without patient-level or clinical outcomes, and nonprimary literature such as conference abstracts, editorials, letters, commentaries, case reports, or reviews were excluded. Non-English-language manuscripts were also omitted.

All titles and abstracts were screened with Rayyan, a free web-based application, with discrepancies being resolved through discussion. The following data were extracted: title, authors, year of publication, study design, number of patients, age group (adult or pediatric), country, aim, algorithm/model, traditional risk model comparison, number of variables, top 3 contributing variable, surgery, outcome, interpretability tool, the best algorithm, and method of validation. The method of validation reported in each study was categorized into 1 of 3 groups: external validation, internal validation (including hold-out test sets and cross-validation), or not provided. These categories were used to descriptively synthesize the rigor of model validation across included studies.

Given the expected clinical and methodological heterogeneity in AI applications across cardiac surgery, a narrative and descriptive quantitative synthesis approach was adopted. Studies were grouped according to AI application and clinical outcome predicted. For performance outcomes, we extracted standardized metrics, including area under the receiver operating characteristic curve (AUC), accuracy, sensitivity, and specificity, where available. AUC is a commonly used metric that evaluates a model’s overall performance in differentiating between distinct groups. An AUC of 0.5 reflects performance equivalent to random chance, whereas 1.0 indicates perfect discrimination. AUC values between 0.7 and 0.8 are generally considered fair, and those between 0.8 and 0.9 are regarded as good.^6^ When multiple metrics were reported, AUC was prioritized for its comparability across classification thresholds. Findings were synthesized narratively and descriptively, with study characteristics and results tabulated for each AI application.

## Results

**Fig. 1.**
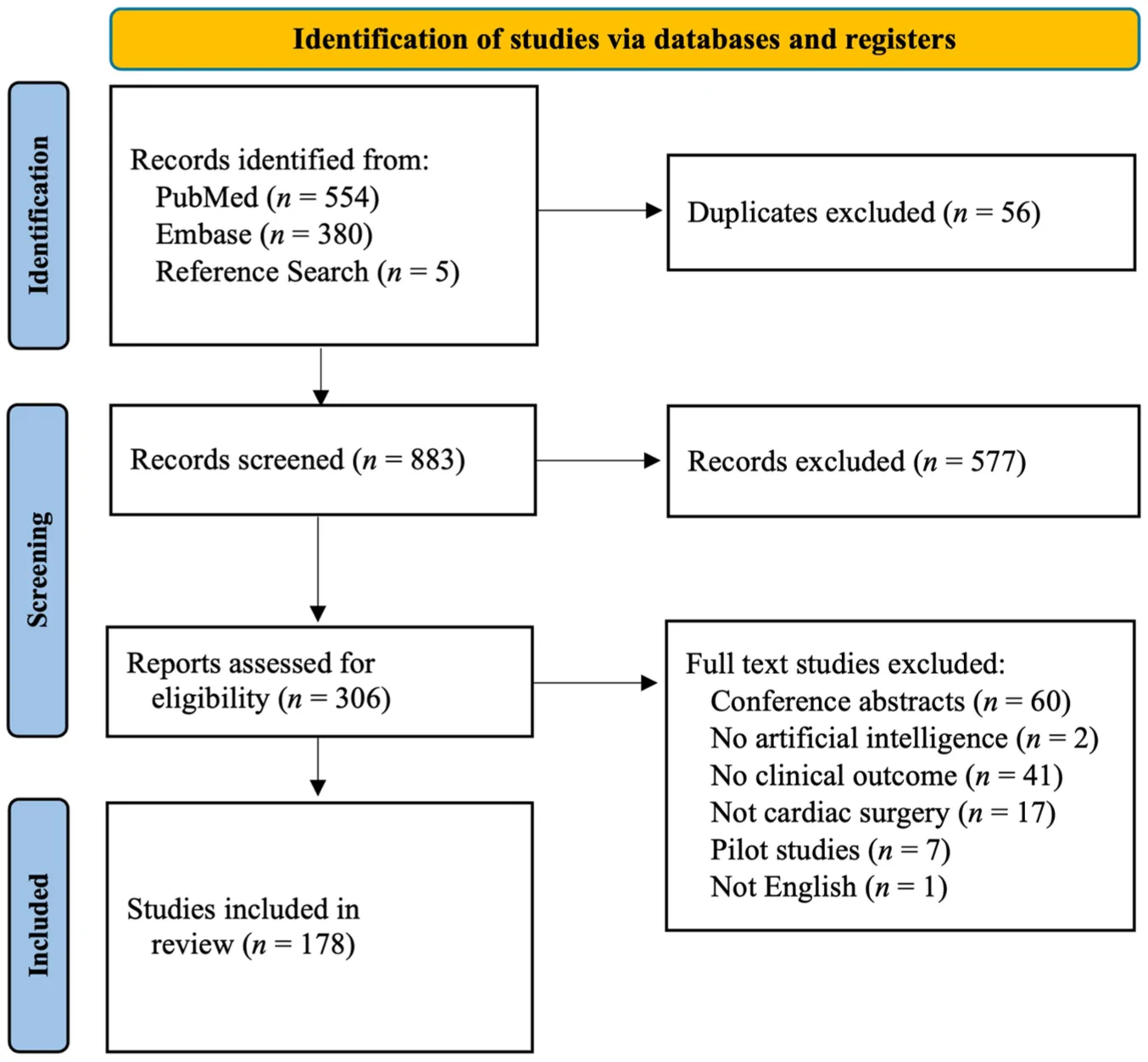
PRISMA flow diagram showing the study selection process.

A total of 939 studies were identified through the literature search, including 554 from PubMed and 380 from Embase. After removing 56 duplicates, 883 studies remained for screening. Of these, 577 were excluded based on title and abstract screening, leaving 306 studies for full-text eligibility assessment. An additional 5 studies were identified through reference searching. In total, 178 studies met the inclusion criteria and were included in this review (Supplemental Appendix). Figure 1 presents the PRISMA flow diagram.

A total of 178 studies were included, distributed by publication year as follows: 2020 (*n* = 9, 5%), 2021 (*n* = 33, 19%), 2022 (*n* = 32, 18%), 2023 (*n* = 29, 16%), 2024 (*n* = 48, 27%), and 2025 (*n* = 27, 15%). The majority of studies employed a retrospective cohort design (*n* = 168, 94%). Most of the studies were conducted in the United States (*n* = 62, 35%), China (*n* = 52, 29%), or Germany (*n* = 13, 7%). Table 1 summarizes the geographical distribution of the included studies. The patient populations were predominantly adults (*n* = 165, 92%), with pediatric populations represented in 13 studies (7%). The most commonly used databases were The Society of Thoracic Surgeons (STS) Adult Cardiac Surgery Database (*n* = 11) and the Medical Information Mart for Intensive Care (*n* = 9). The most frequently used ML models were random forest (*n* = 82, 46%), logistic regression (*n* = 82, 46%), eXtreme Gradient Boosting (*n* = 70, 39%), and support vector machines (*n* = 48, 27%). Regarding the study purposes, 102 (57%) aimed to predict postoperative complications or morbidity, 70 (39%) focused on mortality prediction, 18 (10%) identified disease markers or predictors, 6 (3%) studied resource use, and 4 (2%) developed decision support tools. Supplemental Table 1 presents the study characteristics.

**Table 1.** Geographical Distribution of the Included Studies.

Country	Number of studies
United States of America	62
China	52
Germany	13
France	5
United Kingdom	4
Greece	4
Australia	4
Netherlands	4
Italy	4
South Korea	3
Iran	2
Switzerland	2
Taiwan	2
Brazil	2
Poland	2
Ethiopia	2
Austria	1
Saudi Arabia	1
Spain	1
Lithuania	1
Colombia	1
India	1
Czech Republic	1
Canada	1
Portugal	1
Turkey	1
Serbia	1
Israel	1
Japan	1
Norway	1

Among the 102 studies predicting complications, the most commonly assessed outcomes were acute kidney injury (AKI; *n* = 24), stroke (*n* = 13), and reoperation (*n* = 10). Of the studies predicting AKI, most (*n* = 22) used the KDIGO definition of AKI.^7^ More than half (*n* = 15) predicted AKI of any severity, whereas 8 targeted stage 2 to 3 AKI and 1 focused on stage 3 specifically. Preoperative serum creatinine (*n* = 10) was the most frequent top predictor for AKI, followed by preoperative estimated glomerular filtration rate (*n* = 5), age (*n* = 4), and intraoperative urine output (*n* = 3). Supplemental Table 2 presents the results of the included studies.

A total of 70 studies predicting mortality following cardiac surgery were identified. Mortality was predicted across varying time frames, ranging from in-hospital outcomes to as long as 5 years after surgery. Nine studies compared their model performance against the STS risk scores, of which all studies showed ML models achieved similar if not superior performance. Four benchmarked against European System for Cardiac Operative Risk Evaluation (EuroSCORE) I and 10 against EuroSCORE II. All studies demonstrated superior performance from the ML models again. Benedetto et al. found that retrained logistic regression and random forest achieved an AUC of 0.80 (95% confidence interval [CI]: 0.77 to 0.83) and an AUC of 0.80 (95% CI: 0.76 to 0.83) when predicting in-hospital mortality, compared with EuroSCORE I and II (AUC = 0.76, 95% CI: 0.73 to 0.79 and AUC = 0.77, 95% CI: 0.70 to 0.84).^8^ Other clinical risk models, such as MitraScore and IMPACT, were also evaluated. The top predictors were age (*n* = 22), preoperative creatinine levels (*n* = 17), and ejection fraction (*n* = 7).

Adherence to methodological best practices varied considerably across studies. Only 47 studies (26%) included external validation, with most relying solely on internal validation methods such as cross-validation or hold-out test sets. Reporting standards were also suboptimal: only 34 studies (19%) adhered to TRIPOD or TRIPOD-AI guidelines. Furthermore, 47 studies (26%) did not report the number of parameters or features used in their final models. Code availability was rarely reported, with few studies providing open-source implementations, shared model weights, or end-to-end reproducible pipelines. Most relied solely on internal validation methods such as cross-validation or hold-out test sets (*n* = 111, 62%), whereas 20 studies (11%) did not provide sufficient information to determine the method of validation used. These limitations have important implications for reproducibility, generalizability, and clinical translation of the reported models.

A major challenge in applying ML in clinical settings is its “black box” nature, where the decision-making process is opaque. It is unknown how and why the model arrives at a particular prediction. This lack of transparency limits understanding of which features influence predictions and hampers trust in model outputs. Tools such as SHapley Additive exPlanations (SHAP) have been developed to improve interpretability by illustrating each feature’s positive or negative contribution to the model’s prediction. SHAP was applied in 66 studies, whereas other interpretability tools such as Local Interpretable Model-Agnostic Explanations and permutation feature importance were also used.

## Discussion

AI has shown promising results in cardiac surgery by predicting patient outcomes, identifying risk factors for adverse events, and optimizing resource use. Ensemble tree-based models such as random forest and eXtreme Gradient Boosting are among the most commonly used models. Despite its limited capacity to model complex nonlinear interactions without feature engineering, logistic regression is still commonly used. More advanced or complex ML algorithms do not necessarily result in superior predictive ability. For instance, Gomes et al. showed that despite the higher complexity, a fully connected neural network performed worse than random forest models in predicting in-hospital mortality following transcatheter aortic valve implantation.^9^ This suggests that increased model complexity alone does not guarantee better performance, potentially due to overfitting associated with a large number of trainable parameters, particularly in relatively small clinical datasets.

The type of variables used has a great effect on the performance of the ML model. Including routinely collected postoperative variables has been shown to be associated with improved prediction.^10^ Compared with including only preoperative variables, models that also incorporate intraoperative variables have mixed performance. Castela Forte et al. have shown inferior performance after including 12 routinely collected intraoperative continuous data, such as time series of hemodynamics, temperature, and cardiopulmonary bypass perfusion measurements, when predicting 5-year mortality.^11^ On the contrary, Mori et al. reported a sizable increased performance in operative mortality and 6 postoperative complications when including 96 intraoperative variables.^12^ Similarly, both Luo et al. and Song et al. demonstrated substantial improvements in predictive accuracy by including intraoperative data in predicting cardiac surgery–associated AKI.^13,14^ This could be due to the nature of the intraoperative variables. Mori et al. used intraoperative data on interventions, such as blood transfusion and the use of intra-aortic balloon pump (IABP), as well as time series data. Intraoperative interventions may better reflect the hemodynamic status of the patient. In fact, plasma transfusion followed by any transfusion and IABP usage were identified as the top 3 predictors in the same study, proving their importance over other intraoperative data. Nonetheless, intraoperative variables consistently emerge among the most important predictors, highlighting that the risks of postoperative complications and mortality are influenced not only by preoperative patient profiles but also significantly by pathophysiological changes occurring during surgery. Although there are clear benefits to including these variables, limiting models to preoperative variables may enhance their practicality and usability in real-world clinical scenarios, particularly for preoperative risk assessments.

However, an increased number of variables used for training ML models does not automatically enhance predictive capability.^15^ This could be due to the introduction of noise when more variables are added, potentially degrading model performance. On one hand, a larger feature set could add practical challenges in clinical settings, reducing the applicability and usability of the model; on the other hand, a small feature set might risk omitting potentially important predictors. Hence, there is a fine balance when selecting variables to include when optimizing the model’s performance. In addition, the nature and prevalence of an outcome affects the accuracy of a predictive model. Rarer events are less predictable than more common events. For example, mortality is easier to predict than permanent stroke. This may be a result of overfitting, where the model learns the training data too well and thus not performing on test data. This results in it underestimating the probability of an outcome in low-risk patients and overestimating it in high-risk patients. In rare events, the nature of the event has a greater effect on model performance. Deep sternal wound infection is easier to predict than permanent stroke even though it is rarer. This could be due to the former having a clearer pattern of predictors, with specific diabetes-related variables, such as hemoglobin A1c value and insulin usage.^16^

Currently, postoperative adverse events are estimated using clinical risk models. There were 40 (22%) studies identified in this systematic review that compared ML models with those traditional models. With regard to postoperative AKI, most of the traditional risk models such as the Cleveland Clinic score predict only severe AKI cases requiring renal replacement therapy, which are relatively rare.^17^ In terms of mortality, traditional risk models such as the EuroSCORE have been shown to overestimate the actual risk.^18^ They are also based on logistic regression models of already identified risk factors, which may omit other potential risk factors and their interactions. Elmahrouk et al. identified factors not included in EuroSCORE II that are predictive of mortality in patients undergoing CABG, such as hypertension, shock, troponin level, and atrial fibrillation.^19^

Although several ML models demonstrated performance comparable with, rather than markedly superior to, established clinical risk stratification scores, noninferiority does not necessarily preclude clinical utility. AI-based models may offer advantages beyond discrimination metrics alone, including automation of risk estimation, scalability across large patient populations, and integration into electronic health record systems without additional clinician workload.^20^ Unlike fixed traditional risk scores, ML models can be recalibrated or retrained to reflect local patient populations, evolving surgical techniques, and changes in perioperative care, which is particularly important because traditional risk models tend to deliver similar performance on new cohorts only when those cohorts closely resemble the populations on which the models were originally trained. Newer ML models such as random forest have the advantage of being able to adapt to new patients included. With the superior predictive performance shown by ML models, they can provide clinicians with more accurate risk stratification in preoperative assessments.

However, AI-based prediction models should be interpreted within the context of established cardiac surgery risk scores, particularly the STS risk calculator and EuroSCORE II. These widely used tools are based on logistic regression and large, high-quality registry datasets, and they continue to be updated to reflect contemporary practice. Many studies included in this review used logistic regression as an ML method or benchmarked performance against STS and EuroSCORE models, underscoring the relevance of these traditional approaches within the broader AI landscape. The ongoing development of updated models, including EuroSCORE 3, highlights that methodological rigor and continuous recalibration remain as important as algorithmic complexity.^21^ Consequently, any incremental predictive gains offered by newer AI-based models must be weighed not only against these established benchmarks but also against their practical implications for clinical deployment. New AI models must therefore demonstrate clear advantages over these established tools to justify clinical adoption.

In this context, whether these potential advantages translate into meaningful cost-effectiveness remains unclear. Few studies have evaluated implementation costs, workflow impact, or downstream effects on clinical decision-making and patient outcomes. As such, prospective impact studies and formal health economic evaluations are required to determine whether investment in AI-based risk prediction systems is justified over simpler, established risk stratification methods.

This systematic review has several important limitations. Most studies (64%, *n* = 114) were conducted in the United States or China, potentially limiting the generalizability of findings to other populations. In addition, only 26% of studies (*n* = 47) included external validation, which raises concerns about the robustness and broader applicability of the predictive models. Reliance on internal validation alone risks overestimating predictive performance and limits confidence in generalizability across patient populations. The proliferation of internally validated models without robust external testing may therefore contribute to research redundancy rather than meaningful clinical progress. In parallel, adherence to reporting standards was also poor. Only 19% of studies (*n* = 34) complied with the TRIPOD or TRIPOD-AI guidelines,^5^ reflecting limited transparency in model development and validation. Inadequate reporting is a well-documented problem in prediction model studies, and this hampers reproducibility and peer appraisal.^22^ This issue is particularly concerning given the increasing complexity of AI and ML methods. The recently published TRIPOD-AI guideline was developed to address these challenges by providing specific guidance for reporting AI-based prediction models, including details on data preprocessing, model training, hyperparameter selection, and validation strategy. Wider adoption of TRIPOD-AI is essential to improve reproducibility, enable meaningful comparison across studies, and ensure high-fidelity AI research in cardiac surgery.

A further limitation relates to incomplete reporting of model parameters. In this review, 47 studies (26%) did not report the number of parameters or features used in their final models. This omission has important implications for reproducibility, interpretability, and clinical translation. Without clear reporting of model dimensionality, it is difficult to assess the risk of overfitting, compare model complexity across studies, or understand whether performance gains arise from genuinely informative predictors or from excessive model flexibility. In particular, for tree-based and ensemble methods, the absence of information on feature counts, tree depth, or regularization parameters limits meaningful comparison between models that may differ substantially in complexity despite reporting similar performance metrics. From a clinical perspective, poor reporting of model parameters also hampers implementation. Models that rely on a large number of variables may be impractical to deploy in real-world settings, especially if those variables are not routinely collected or require extensive preprocessing. Conversely, parsimonious models with comparable performance may offer greater usability and robustness but cannot be identified when parameter reporting is incomplete. Although most studies employed standard ML algorithms implemented in widely available open-source libraries, reproducibility was often limited by incomplete reporting of implementation details. Few studies explicitly reported code availability, shared trained models, or provided end-to-end pipelines encompassing data preprocessing, feature engineering, model training, and evaluation. As a result, independent reproduction and external benchmarking of published models remain challenging, even when commonly used algorithms are employed. In addition, key performance metrics such as sensitivity were often underreported, potentially overstating model effectiveness and clinical relevance. Lastly, publication bias may exist, as studies with positive outcomes are more likely to be published, possibly skewing perceptions of AI model performance.

This review has implications for both AI researchers and clinicians. For researchers, the findings underscore the need to prioritize external validation, transparent reporting, and prospective evaluation over incremental improvements in discrimination metrics. Methodological rigor, adherence to TRIPOD-AI, and reproducibility are likely to have a greater impact on clinical translation than increasingly complex model architectures. For clinicians considering the adoption of AI-based risk prediction tools, careful appraisal of methodological quality is essential. Beyond headline performance metrics such as AUC, clinicians should evaluate whether a model has undergone external validation in populations similar to their own and whether calibration and clinically relevant performance measures are reported. Clear specification of the intended clinical use case, such as preoperative risk stratification or postoperative complication monitoring, is also critical. Models that integrate seamlessly into existing workflows, provide interpretable outputs, and demonstrate performance comparable to or exceeding established risk scores are more likely to offer real-world utility. Without these attributes, even high-performing models may fail to influence clinical decision-making.^23^

## Conclusions

AI models show strong potential to enhance risk prediction in cardiac surgery, outperforming traditional clinical tools in predicting complications such as AKI and mortality. Tree-based models such as random forest and eXtreme Gradient Boosting frequently delivered high performance, often using standard preoperative and intraoperative variables. However, the current evidence base is limited by retrospective designs, infrequent external validation, poor adherence to reporting standards, and limited reproducibility. Greater alignment with TRIPOD-AI guidelines, increased transparency, and prospective multicenter validation are required before widespread clinical implementation can be recommended. Future research should focus not only on predictive performance but also on clinical integration, interpretability, and real-world impact.

## Supplemental Material

sj-docx-1-inv-10.1177_15569845261457046 – Supplemental material for Artificial Intelligence for Predicting Perioperative Outcomes in Cardiac Surgery: A Systematic Review Supplemental material, sj-docx-1-inv-10.1177_15569845261457046 for Artificial Intelligence for Predicting Perioperative Outcomes in Cardiac Surgery: A Systematic Review by Jason Ha and Hunaid A. Vohra in Innovations
