# Supplementary material for: Artificial Intelligence for Predicting Perioperative Outcomes in Cardiac Surgery: A Systematic Review
Source: Innovations (Phila). 2026 Jul 13;21(3):210–6. doi: 10.1177/15569845261457046 (PMC13534759; doi:10.1177/15569845261457046)
Supplement: Supplementary file 1 [file sj-docx-1-inv-10.1177_15569845261457046.docx]

**Supplemental Table 1.** Characteristics of Included Studies.

| **#** | **Reference** | **Design** | **Aim** | **Country** | **Population** | **AI model** | **Number of variables** | **Interpretability** | **Method of validation (internal/external)** |
| --- | --- | --- | --- | --- | --- | --- | --- | --- | --- |
| 1 | Abbasi et al. (2025) | Retrospective cohort study using STS ACSD | To predict hemorrhage requiring reoperation, VTE, and stroke | USA | 662,772 adults undergoing cardiac surgery | DL (FFNN) | 240 | SHAP, permutation feature importance | Internal - 80% training, 10% testing, 10% validation |
| 2 | Abdul Ghffar et al. (2020) | Retrospective cohort study | To predict in-hospital and 30-days all cause and cardiovascular mortality | USA | 344 adults undergoing TAVI | TDA + semi-supervised AutoML (OptiML, DT, LR, DNN) | 23 | NP | Both - 70% training, 20% validation. Monte Carlo CV applied within the training set |
| 3 | Agasthi et al. (2020) | Retrospective cohort study using ISHLT registry | To predict 5-year mortality and graft failure | USA | 15,236 adults undergoing orthotopic heart transplantation | GBM | 87 | NP | Internal - 80% training, 20% validation. 10-fold CV repeated 5 times on training set |
| 4 | Agasthi et al. (2021) | Retrospective cohort study | To predict 1-year mortality | USA | 1,055 adults undergoing TAVI | GBM | 183 | Feature importance | Internal - 5-fold CV repeated 10 times |
| 5 | Alhwiti, Aldrugh and Megahed (2023) | Retrospective cohort study using NIS/HCUP database | To predict in-hospital mortality | USA | 54,739 adults undergoing TAVI | SVM, KNN, DT, GBC, LGBM, CatBoost, AdaBoost, XGBoost, RF, ExtraTrees, LR, Ridge Regression, LDA, QDA, NB | 5, 10, 20, 30, 40, 45 | Feature importance | Internal - 80% training, 20% validation. 5-fold CV |
| 6 | Allou et al. (2023) | Retrospective cohort study using Epicard database | To predict in-hospital mortality | France | 165,640 adults undergoing cardiac surgery | TabBERT (DL), RF, XGBoost | NP | SHAP | Internal - 80% training, 20% testing, 5-fold CV |
| 7 | Arabyarmohammadi et al. (2024) | Retrospective cohort study | To predict severity of cardiac allograft rejection | USA | 2,900 biopsy samples | Multivariable LR | 370 | Feature importance | Internal - 3-fold CV repeated 500 times |
| 8 | Aranda-Michel et al. (2022) | Retrospective cohort study | To evaluate relationship between MELD and outcomes of cardiac surgery | USA | 3,872 patients undergoing cardiac surgery | RF, RSF, ENR, LR | NP | Feature importance | Internal - 63.2% training, 26.8% out-of-bag error estimation and validation |
| 9 | Arian et al. (2022) | Retrospective cohort study | To predict myocardial function improvement | Iran | 43 patients undergoing CABG | SCAD-SVM, RP | 93 | Feature importance | Internal - 5-fold CV repeated with 10,000 bootstrapping |
| 10 | Ayers et al. (2021) | Retrospective cohort study using UNOS registry | To predict 1-year mortality | USA | 33,657 adults undergoing orthotropic heart transplantation | Ensemble (RF, DNN, LR, AdaBoost) | 47 | Feature importance | Internal - 80% training, 20% validation |
| 11 | Bartkowiak et al. (2024) | Retrospective cohort study | To predict outcome (mortality, heart failure hospitalization, or tricuspid reoperation throughout two years) | Switzerland | 33 patients undergoing T-TEER | DL (Heart.ai for automated CT analysis) | 49 | NP | NP |
| 12 | Behnoush et al. (2023) | Retrospective cohort study using Tehran Heart Centre CABG registry | To predict 1-year mortality | Iran | 8,493 hypertensive patients undergoing CABG | LR, RF, XGBoost, ANN, NB | 11 | Feature importance | Both - 70% training, 30% testing, 10-fold validation and temporal validation |
| 13 | Benedetto et al. (2020) | Retrospective cohort study using NACSA dataset | To predict in-hospital mortality | UK | 28,720 adults undergoing cardiac surgery | NN, RF, NB, LR | 17 | NP | Both - 70% training, 30% testing, 5-fold CV for hyperparameter tuning, temporal validation |
| 14 | Bertsimas et al. (2021) | Retrospective cohort study using ECDB | To predict mortality, prolonged postoperative mechanical ventilatory support and prolonged hospital stay | Greece | 235,000 patients undergoing congenital cardiac surgery | OCT, RF, GB, LR | 13 | Feature importance | Both – training/testing split and temporal validation |
| 15 | Bertsimas et al. (2022) | Retrospective cohort study using ECDB | To define benchmarking standards after congenital cardiac surgery, permitting case-adjusted hospital-specific performance evaluation | Greece | 64,202 patients undergoing congenital cardiac surgery | OCT | 12 | Feature importance | Both – training/testing split with temporal validation |
| 16 | Betts et al. (2023) | Retrospective cohort study using ANZCORS database | To predict 30-day mortality | Australia | 14,343 pediatric patients undergoing pediatric cardiac surgery | GBT, LR with ENR, ANN | 47 | SHAP | Both – 70% training, 30% testing, 5-fold CV |
| 17 | Bi et al. (2022) | Retrospective cohort study using eICU Collaborative Research Database | To predict in-hospital mortality | USA | 5,860 adults undergoing cardiovascular surgery | XGBoost, RF, DNN, LassoLR, BackwardStepwiseLR | 21 | Feature importance | Internal – 70% training, 30% validation |
| 18 | Bodenhofer et al. (2021) | Retrospective cohort study | To predict 30-day mortality | Austria | 2,229 adults undergoing valvular surgery | RF, ANN, PSVM, LR | 137 | Feature importance | Internal – 5-fold CV |
| 19 | Boskovski et al. (2020) | Retrospective cohort study | To evaluate the impact of de novo damaging variants on postoperative outcomes | USA | 2,517 patients with congenital heart disease | Model-free ML for high-risk variant discovery | NP | NP | NP |
| 20 | Cai et al. (2021) | Retrospective cohort study | To predict surgery-related pressure injury | China | 149 patients undergoing cardiovascular surgery | XGBoost, LR | 9 | Feature importance | NP |
| 21 | Castela Forte et al. (2022) | Retrospective cohort study using CAROLA registry | To predict 30-day, 1-year, 5-year mortality | Netherlands | 9,415 patients undergoing first-time cardiac surgery | LSTM, GBT (XGBoost) | 12 | SHAP | Internal – 80% training, 20% validation |
| 22 | Castela Forte et al. (2021) | Retrospective cohort study using CAROLA registry | To predict 5-year mortality | Netherlands | 8,241 patients undergoing cardiac surgery | Super Learner (BART, ExtraTrees, ENR, SVM, XGBoost) | 88 | Feature importance | Internal – 10-fold CV |
| 23 | Che et al. (2024) | Retrospective cohort study | To predict intraoperative red blood cell transfusion volume | China | 4,285 patients undergoing aortic surgery | ExtraTrees, RF, XGBoost, CatBoost | 70 | SHAP | Internal – 70% training, 30% testing |
| 24 | Chen et al. (2022) | Retrospective cohort study | To predict ventilator weaning success within 24 hours | Taiwan | 1,439 patients in cardiac intensive unit | SVM, LR, XGBoost, DT, RF, ANN | 7 | Feature importance | Internal – 10-fold CV |
| 25 | Chen et al. (2024) | Retrospective cohort study | To predict AKI of all stages for longer than 2 days | China | 5,368 patients undergoing cardiac surgery | LR | 9 | Nomogram | Internal – 70.2% training, 29.8% validation |
| 26 | Chen et al. (2020) | Multicenter prospective study | To predict severe AKI (stage 2-3) | China | 402 adult patients undergoing cardiac surgery | LR | NP | Nomogram | NP |
| 27 | Couto et al. (2024) | Retrospective study | To predict hospital length of stay | Brazil | 9,584 adult patients undergoing cardiac surgery | RF, XGBoost, NN | 59 | SHAP | Both – 10-fold CV and temporal validation |
| 28 | Cruz et al. (2024) | Retrospective study using NRD | To predict in-hospital morality | USA | 2,450 adult patients undergoing TMVR | LR, RF, XGBoost, KNN, NB | 21 | SHAP | Internal – 3-fold CV repeated 20 times |
| 29 | Cruz et al. (2024) | Retrospective study using NRD | To predict hospitalization costs | USA | 444,740 adult patients undergoing cardiac surgery | XGBoost, RF, KNN, LR | NP | SHAP | Internal – 75% training, 25% validation, 10-fold CV |
| 30 | da Cunha et al. (2024) | Retrospective cohort study | To predict the need for blood transfusion | Brazil | 495 patients undergoing cardiac surgery | LR, RF, SVM, MLP | 6 | Permutation feature importance | Internal – 80% training, 20% testing, k-fold CV |
| 31 | da Cruz et al. (2021) | Retrospective cohort study using MIMIC-III database | To validate an existing model on predicting AKI | Germany | 25,799 patients undergoing cardiac surgery | GBDT, RF, LR, EN, AdaBoost, DT | 40 | SHAP | Both – 10-fold CV on unseen cohort |
| 32 | Dai et al. (2023) | Retrospective cohort study | To predict AKI of any severity | China | 265 patients undergoing type A acute aortic dissection repair | LR, RF, XGBoost, SVM | 9 | SHAP | Internal – 80% training, 20% testing, 10-fold CV |
| 33 | Del Gaizo et al. (2024) | Retrospective cohort study | To predict morbidity and mortality (postoperative stroke, acute renal failure, cardiac reoperation, deep sternal wound infection, and prolonged ventilation) | USA | 1,738 patients undergoing CABG | TextCNN (AttnToNum, ScaleNum), TextLSTM | NP | SHAP-N | Internal – 85% training, 15% validation |
| 34 | Dong et al. (2025) | Retrospective cohort study using MIMIC-IV database | To predict gastrointestinal bleeding | China | 16,440 patients undergoing CABG | MLP, NB, RF, XGBoost, LR, SVM | 15 | SHAP | Both – 5-fold CV |
| 35 | Dong et al. (2024) | Retrospective cohort study using NACSA database | To predict 30-day mortality | UK | 227,087 patients undergoing cardiac surgery | LR, XGBoost, BME | 61 | NP | Both – temporal holdout validation |
| 36 | Duong et al. (2022) | Retrospective cohort study | To identify patients with postoperative heart block who are at high risk of requiring permanent pacemaker implantation | USA | 139 paediatric patients undergoing congenital heart surgery | DT | NP | NP | Both – temporal validation |
| 37 | Elmahrouk et al. (2024) | Retrospective cohort study using Gulf Left Main Registry | To predict 30-day mortality | Saudi Arabia | 866 adult patients undergoing CABG for LMCA disease | LR, RF, KNN, SVM, NB, MLP, AdaBoost, CAT, LGBM, XGBoost | NP | SHAP, QLattice | Internal – 70% training, 30% testing |
| 38 | Evertz et al. (2022) | Retrospective cohort study | To predict cardiovascular mortality | Germany | 142 patients undergoing TAVI | Commercial AI software (suiteHEART® Neosoft) | NP | NP | NP |
| 39 | Fan et al. (2022) | Retrospective cohort study | To predict mortality (STS definition) | China | 5,443 adult patients undergoing cardiac surgery | RF, NN, SVM, GBM, LR | NP | SHAP | Internal – 80% training, 20% testing, 10-fold CV on training |
| 40 | Fan et al. (2023) | Retrospective cohort study | To predict AKI of any severity | China | 778 adult patients undergoing cardiac surgery with CPB | LR, RF, DeepForest, XGBoost | 43 | SHAP | Both – temporal validation |
| 41 | Fernandes et al. (2021) | Retrospective cohort study using STS database | To predict mortality (STS definition) | USA | 5,015 adult patients undergoing cardiac surgery | LR, RF, NN, SVM, XGBoost | NP | NP | Internal – 75% training, 25% testing |
| 42 | Fliegenschmidt et al. (2022) | Retrospective cohort study using a quality assurance program database | To predict postoperative delirium | Germany | 114 adult patients undergoing cardiac surgery | Transformer-based DL integrated with hospital HER | 56 | LIME | Internal – 80% training, 20% testing |
| 43 | Fragasso et al. (2023) | Retrospective cohort study | To predict AKI of any severity and stage 2-3 after 48 hours | Italy | 419 pediatric patients in cardiac intensive unit | RF | 36 | Feature importance plot | Internal – 70% training, 30% testing |
| 44 | Gao et al. (2023) | Retrospective cohort study | To predict heparin resistance | China | 296 pediatric patients undergoing congenital cardiac surgery with CPB | LR | 35 | Nomogram | Internal – 70% training, 30% testing |
| 45 | Giangreco et al. (2021) | Retrospective cohort study | To identify biomarkers and predictors for primary graft dysfunction | USA | 88 patients undergoing heart transplantation | LR | 181 | Feature importance | Both – independent cohort of 65 patients |
| 46 | Gomes et al. (2021) | Retrospective cohort study | To predict all-cause mortality, stroke, major vascular complications, paravalvular leakage, and new pacemaker implantations | Germany | 451 patients undergoing TAVI | RF, SVM, ANN | 83 | Feature importance | Internal - five-fold nested CV |
| 47 | Griffeth et al. (2024) | Retrospective cohort study | To predict operative mortality, mechanical circulatory support, dialysis, unplanned noncardiac reoperation, persistent neurologic deficit, and cardiac arrest | USA | 1,011 adults undergoing congenital cardiac reoperation | XGBoost, LR | 7 | SHAP | Internal – 70% training, 20% testing, 10% validation. Bootstrapping with 1000 iterations, 5-fold CV |
| 48 | Habertheuer et al. (2021) | Retrospective cohort study using STS ACSD | To predict operative mortality and morbidity (reintubation, prolonged ventilation, pneumonia, renal failure, dialysis, stroke, reoperation for bleeding, and a permanent pacemaker) | USA | 1,181 adults underwent cardiac surgery for complications related to IE | LR | 7 for mortality model, 7 for morbidity model | NP | Internal - 80% training, 20% validation, 5-fold CV on training set |
| 49 | Halloran et al. (2024) | Retrospective cohort study | To distinguish rejection states and their relationship to injury and graft survival | USA | 3,230 patients undergoing heart transplantation | MMDx (archetypal analysis + ensemble ML classifiers) | NP | NP | NP |
| 50 | Haregu et al. (2025) | Retrospective cohort study using OPTN database | To predict waitlist mortality | USA | 5,523 pediatric patients undergoing heart transplantation | Lasso, GAM, XGBoost, RF, CatBoost | 42 | SHAP | Internal – 82% training, 18% testing |
| 51 | Hausleiter et al. (2024) | Retrospective cohort study using EuroSMR registry | To predict 1-year mortality | Germany, Italy, Spain, France | 4,600 patients undergoing M-TEER | XGBoost | 18 | SHAP | Both – 5-fold CV, validation on 2 independent sites |
| 52 | Hui et al. (2023) | Retrospective cohort study using ANZSCTS database | To predict postoperative bleeding (Papworth and Dyke definition) | Australia | 2,000 adult patients undergoing cardiac surgery with CPB | LR, RF, XGBoost, DNN, Ensemble Voting Classifier | 20 | SHAP | Internal – 80% training, 20% testing |
| 53 | Hur, Wi and Kim (2020) | Retrospective cohort study using MIMIC-III database | To predict unplanned cardiac surgery | USA | 11,811 adult patients with heart disease | LSTM attention model | 251 | Heat map | Internal – 70% training, 15% validation, 15% testing |
| 54 | Jia et al. (2023) | Retrospective cohort study | To predict AKI of any severity | China | 4,831 adult patients undergoing CABG | LGBM, SVM, RF, Softmax | 26 | SHAP | Both – 80% training, 20% validation, 2 independent sites |
| 55 | Jiang et al. (2025) | Retrospective cohort study using MIMIC-IV database | To predict extubation failure | China | 776 adult patients undergoing cardiac surgery | XGBoost, RF, LR | NP | SHAP | Internal – 80% training, 20% testing, 5-fold CV on training |
| 56 | Jiang et al. (2023) | Retrospective cohort study | To predict AKI of any severity, and stage 2-3 | China | 2,310 adult patients undergoing cardiac surgery | LR, DT, RF, GBC, NB, MLP | 16 | SHAP | Internal – 80% training, 20% validation |
| 57 | Kachroo et al. (2022) | Retrospective cohort study | To predict repair durability using STS database variables | USA | 224 patients undergoing surgical revascularization and mitral valve repair | SVM, LR, DNN | 53 | Feature importance | Internal – 80% training, 20% testing |
| 58 | Kageyama et al. (2024) | Retrospective cohort study | To predict 5-year mortality | Poland | 1,035 patients undergoing revascularization (356 CAGB, 679 PCI) | LASSO | 42 | NP | Internal – 10-fold CV |
| 59 | Kainuma et al. (2022) | Retrospective cohort study using United Network for Organ Sharing registry | To identify predictors of 1-year mortality | USA | 1,294 adults underwent cardiac re-transplantation | RSF | NP | Feature importance | NP |
| 60 | Kalisnik et al. (2022) | Retrospective cohort study | To predict AKI of any severity | Germany | 7,507 patients undergoing cardiac surgery | XGBoost, LR, RF, SVR, DNN | 85 | SHAP | Internal – random split, 10-fold CV |
| 61 | Kampaktsis et al. (2023) | Retrospective cohort study using UNOS database | To predict 1-year mortality | USA | 1,033 adult patients undergoing heart transplantation | CatBoost | 10 | SHAP | Internal – 70% training, 30% validation |
| 62 | Kampaktsis et al. (2021) | Retrospective cohort study using UNOS database | To predict 1-year, 3-year, 5-year mortality and acute rejection | USA | 18,625 adult patients undergoing heart transplantation | AdaBoost, LR, DT, SVM, KNN | 39 | LIME | Internal – 3:1 split |
| 63 | Karri et al. (2021) | Retrospective cohort study using MIMIC-III database | To predict postoperative atrial fibrillation | USA | 6,040 adult patients undergoing cardiac surgery | GBM, RF, LR, DT, KNN, SVM | 45 | SHAP | Internal – 5-fold CV repeated 4 times |
| 64 | Kilic, Habib, et al. (2021) | Retrospective cohort study using STS ACSD | To predict operative mortality and morbidity (acute renal failure, prolonged ventilation, reoperation, stroke, and deep sternal wound infection) | USA | 243,142 adults undergoing SAVR | XGBoost | NP | NP | Internal - 80% training, 20% testing |
| 65 | Kilic, Goyal, et al. (2021) | Retrospective cohort study using STS ACSD | To predict operative mortality and morbidity (acute renal failure, prolonged ventilation, reoperation, stroke, and deep sternal wound infection) | USA | 243,142 adults undergoing SAVR | XGBoost | NP | NP | Internal - 80% training, 20% testing. Bootstrapping with 1000 iterations |
| 66 | Kim et al. (2022) | Retrospective cohort study | To predict postoperative cardiovascular deterioration | USA | 423 adult patients undergoing elective cardiac surgery | RF, SVM, NB, LUCCK | NP | SHAP | Both – 3-fold CV, 2 independent cohorts |
| 67 | Kim and Jang (2021) | Retrospective cohort study | To predict bleeding risk after warfarin | South Korea | 598 adult patients undergoing mechanical valve replacement | RF | 22 | Feature importance | NP |
| 68 | Kirchner et al. (2024) | Retrospective cohort study | To predict mortality and hospitalization | Germany | 100 adult patients undergoing TTVR | DL (Heart.ai for cardiac CT segmentation) | NP | NP | NP |
| 69 | Kobayashi et al. (2023) | Retrospective cohort study | To predict maximum postoperative lactate concentration | USA | 2,187 adult patients undergoing cardiac surgery with CPB | LR, RF, ANN, RNN, Transformer | NP | SHAP | Internal – repeated nested 5-fold CV |
| 70 | Kurmanaliyev et al. (2025) | Retrospective cohort study | To predict mortality, stroke, life-threatening bleeding, acute kidney injury (stage 2 or 3), coronary artery obstruction requiring intervention, major vascular complications, and valve-related dysfunction requiring reoperation | Lithuania | 224 patients undergoing TAVI | RF | 77 | SHAP | Internal – 75% training, 25% testing, 10-fold CV |
| 71 | Kwak et al. (2021) | Retrospective cohort study | To identify cardiovascular magnetic resonance markers in aortic stenosis | South Korea | 799 patients undergoing SAVR or TAVI | RSF | 29 | Feature importance | Both – k-fold CV and 100 random replicates, independent validation cohort |
| 72 | Lachmann et al. (2022) | Retrospective cohort study | To assess 2-year all-cause mortality | Germany | 366 patients undergoing TAVI | Unsupervised agglomerative clustering + ANN | NP | NP | NP |
| 73 | Lachmann et al. (2025) | Retrospective cohort study | To predict right ventricular ejection fraction from echocardiographic videos | Germany | 1,154 patients undergoing M-TEER | DL (RVENet, ensemble of spatiotemporal CNNs) | NP | NP | NP |
| 74 | Li et al. (2023) | Retrospective cohort study | To predict in-hospital mortality | China | 2,414 patients with acute coronary syndrome | LR, XGBoost | 20 | SHAP | Internal – 80% training, 20% validation, nested k-fold CV |
| 75 | Li et al. (2023) | Retrospective cohort study | To predict AKI of any severity | China | 6,495 patients undergoing cardiac surgery with CPB | LR, SVM, KNN, NB, DT, RF, GB, XGBoost, LGBM, CAT, AdaBoost, ExtraTrees | NP | SHAP | Both – 80% training, 20% testing, 5-fold CV on training set, validation using MIMIC-IV database |
| 76 | Li et al. (2025) | Retrospective cohort study | To predict postoperative pulmonary complications | China | 100 adult patients undergoing elective cardiac valve surgery | XGBoost, LR, RF, SVM, KNN | 90 | Feature importance | Internal – 5-fold CV |
| 77 | Li et al. (2024) | Retrospective cohort study | To predict postoperative delirium | China | 507 adult patients undergoing cardiac valve surgery with CPB | RF, GBDT, LR, SVM, KNN, NB, Perceptron | 31 | SHAP | Internal – 80% training, 20% testing |
| 78 | Li et al. (2022) | Retrospective cohort study | To predict stage 3 AKI | China | 107 adult patients undergoing heart transplantation | LR, RF, NB, SVM | 6 | NP | Internal – 10-fold CV |
| 79 | Li et al. (2024) | Retrospective cohort study | To predict perioperative myocardial injury | China | 2,983 adult patient undergoing elective cardiac surgery with CPB | LR, SVM, KNN, NB, DT, RF, GB, XGBoost, LGBM, CAT, AdaBoost, ExtraTrees | 60 | SHAP | Both – 80% training, 20% testing, 3 independent sites |
| 80 | Li et al. (2020) | Retrospective cohort study | To predict AKI of all severity + stage 2-3 AKI | China | 5,533 adult patients undergoing cardiac surgery | NB | 27 | NP | Both – 10-fold CV, temporal validation |
| 81 | Li et al. (2025) | Retrospective cohort study | To predict postoperative moderate to severe mitral regurgitation | China | 987 patients undergoing TAVI | RF, GBDT, LR, SVM, DT, XGBoost | 60 | SHAP | Internal – 70% training, 30% testing |
| 82 | Liou et al. (2025) | Retrospective cohort study using SRTR database | To predict 1-year mortality | USA | 7,160 adult patients undergoing heart transplantation | Lasso, ElasticNet, Ridge regression, CoxBoost, XGBoostL, RSF, XGBoostT | 75 | Feature importance | Internal – 80% training, 20% validation, nested 5-repeat, 5-fold CV |
| 83 | Lisboa et al. (2022) | Retrospective cohort study using UNOS and Scandinavian Thoracic Transplantation Database | To predict 1-year mortality | USA | 44,178 adult patients undergoing heart transplantation | PRN, PRN-Lasso, EBMs, IHTSA (DL), LR (IMPACT) | 9 | Nomogram | Both – 84% training, 18% validation, temporal validation |
| 84 | Liu et al. (2024) | Retrospective cohort study | To predict heparin pump speed to optimize anticoagulation therapy | China | 1,080 adult patients undergoing cardiac surgery | Full Connected Layer (self-attention), LR, SVM, XGBoost, LSTM, GRU, FC Layer | 764 | SHAP | Both – 80% training, 20% testing, 5-fold CV, temporal validation |
| 85 | Liu et al. (2022) | Retrospective cohort study | To predict AKI of any severity | USA | 1,803 patients undergoing cardiac surgery | Personalised ML with transfer learning | 1892 | Heatmap | Internal – 5-fold CV |
| 86 | Lu et al. (2023) | Retrospective cohort study | To predict postoperative atrial fibrillation | China | 1,400 adult patients undergoing cardiac surgery with CPB | SVM, GBDT, LR | 43 | Feature importance | Internal – 70% training, 30% testing, 10-fold CV |
| 87 | Luo et al. (2022) | Retrospective cohort study | To predict early mortality | China | 476 patients undergoing valve surgery for infective endocarditis | XGBoost, ensemble (GBDT, LGBM, RF, ExtraTrees), LR | 8 | Permutation feature importance | Both – 3-fold CV, 2 independent sites |
| 88 | Luo et al. (2023a) | Retrospective cohort study | To predict AKI of any severity + stage 2-3 AKI | China | 3,278 pediatric patients undergoing cardiac surgery with CPB | XGBoost, KNN, NB, SVM, RF, NN | 25 | SHAP | Both – 5-fold CV, 2 independent sites |
| 89 | Ma et al. (2021) | Retrospective cohort study using CLIATHVR database | To improve warfarin dose prediction accuracy | China | 19,060 adult patients undergoing valve replacement | FFNN (3-layer architecture) | 8 | NP | Both – 2 independent sites |
| 90 | Mao et al. (2025) | Retrospective cohort study | To assess impact of aortic arch morphology on procedural details and outcomes | China | 1,480 patients undergoing TAVI | DL (nnUNet for segmentation), RF, LR (for predictive modelling) | NP | NP | Both – 10-fold CV, 3 independent centers |
| 91 | Marmagkiolis et al. (2024) | Retrospective cohort study using NIS database | To assess prevalence, safety, cost, and disparities of TEER and TMVR in cancer patients | USA | 7495 adult patients undergoing TMVR or TEER | ML-PSr, BAM-PS | 15 | NP | NP |
| 92 | Mathis et al. (2022) | Retrospective cohort study | To predict postop deterioration | USA | 1,555 adult patients undergoing cardiac surgery with CPB | RF, SVM, NB, LUCCK | 430 | NP | Both – 67% training, 33% validation, 3-fold CV repeated 101 times, temporal validation |
| 93 | Mohammadyari et al. (2024) | Retrospective cohort study | To predict 1 year mortality | Italy | 165 patients undergoing TAVI or SAVR | DeepSurv, RSF | 6 | Feature importance | Internal – 70% training, 30% validation, 3 repeated k-fold CV |
| 94 | Molina et al. (2022) | Retrospective cohort study | To predict operative mortality (death during same hospitalization as surgery or within 30 days of discharge) | Colombia | 2,786 adult patients undergoing cardiovascular surgical procedures | LR, NB, MLP, SVM, RF, GB | 23 | Feature importance | Internal – 80% training, 20% testing |
| 95 | Mori et al. (2021a) | Retrospective cohort study using STS ACSD | To predict 30 days mortality, prolonged ventilation, pneumonia, stroke, reoperation, renal failure, deep wound infection | USA | 378,572 adult hospitalizations for CABG | XGBoost, LR | 47 preoperative, 96 postoperative | Feature importance | Internal – 70% training, 30% testing, 20 iterations |
| 96 | Mustafa et al. (2025) | Retrospective cohort study using National Inpatient Sample database | To predict optimal discharge | USA | 25,874 adult patients undergoing TAVI | ANN, XGBoost, LR | NP | Feature importance | Internal – 70% training, 20% testing, 10% holdout for ANN, 75% training, 25% testing for XGBoost |
| 97 | Nagy et al. (2024) | Retrospective cohort study | To predict stage 2-3 AKI at postoperative day 2 | USA | 402 pediatric patients undergoing cardiac surgery | LGBM | 34 | SHAP | Internal – 80% training, 20% testing, 10-fold CV on training set |
| 98 | Namasivayam et al. (2022) | Retrospective cohort study | To predict all-cause mortality or AVR in aortic stenosis | USA | 1,670 patients with moderate or greater severity of aortic stenosis | Bootstrap lasso regression, Ridge logistic regression | 11 | NP | Both - 80% training, 20% testing, 10 stratified bootstrap splits, independent site |
| 99 | Narayan et al. (2022) | Retrospective cohort study | To evaluate impact of preoperative statin therapy on postoperative respiratory complications | India | 5,638 patients undergoing CABG | RF, LR, SGD, ExtraTrees | 92 | Feature importance | Internal – 80% training, 20% testing |
| 100 | Nistal-Nuño (2022) | Retrospective cohort study using MIMIC-III database | To predict in-hospital mortality | USA | 11,059 patients in coronary care unit and cardiac surgery recovery unit | XGBoost, RF, TE, NB, BN, LR | NP | SHAP | Internal –80% training, 20 testing |
| 101 | Nowakowska et al. (2024) | Retrospective cohort study | To predict depression and postop delirium | Poland | 224 adult patients undergoing CABG with or without valve surgery | RF, GBT, AdaBoost, XGBoost | NP | Feature importance | Internal – 10-fold CV |
| 102 | Okuno et al. (2021) | Prospective cohort study | To predict early cerebrovascular events (strokes, TIA) | Switzerland | 1,492 patients undergoing TAVI | Rare-event autoencoder (DNN) | 63 | NP | Internal – 2/3 training, 1/3 testing, validated on 20% of training set |
| 103 | Orfanoudaki et al. (2022a) | Retrospective cohort study using STS ACSD | To predict operative mortality, prolonged ventilation, renal failure, deep sternal wound infection, permanent stroke | USA | 287,662 adults undergoing MSV | LR, RF, XGBoost, OCT, L-OCT | 89 | NP | Internal – 75% training, 25% testing and validation, k-fold CV, 5 random splits and bootstrapping |
| 104 | El Ouahidi et al. (2024) | Retrospective cohort study | To predict pacemaker implantation | France | 520 adults undergoing TAVI | SVM, LR, XGBoost, RF, GB, MLP, GNB, DT | 22 (8 from CT-scan, 7 from ECG, 2 from TTE, 3 procedure-related, 2 clinical variables) | SHAP | Internal – Nested 10-fold CV with outer 90/10 splits for testing and inner CV for tuning |
| 105 | Ouyang et al. (2024) | Retrospective cohort study | To predict postop mortality using preop ECGs | USA | 45,969 patients with ECG waveform image undergoing procedures | PreOpNet (DL CNN) | NP | Heatmap | Both – 80% training, 10% validation, 10 testing, 2 external sites |
| 106 | Pan et al. (2025) | Retrospective cohort study | To predict early postoperative infection | China | 2,001 adult patients undergoing open heart surgery | XGBoost, RF, SVM, Lasso LR, Single-layer NN | 9 | Feature importance | Both – 10-fold CV, 4 external medical centers |
| 107 | Pei et al. (2025) | Retrospective cohort study using MIMIC-IV database | To predict in-hospital and 360-day all-cause mortality | USA | 3,848 adult patients undergoing cardiac surgery | XGBoost, SVM, AdaBoost, NB, LR, GBM | NP | SHAP | Both – 10-fold CV |
| 108 | Pekař et al. (2024) | Retrospective cohort study using Registry of Patients after TAVI | To predict mortality | Czech Republic | 866 patients undergoing TAVI | AutoMATiCA (UNet for CT segmentation) | NP | NP | NP |
| 109 | Penny-Dimri et al. (2021) | Retrospective cohort study using ANZSCTS database | To predict AKI and AKI needing renal replacement therapy | Australia | 96,653 patients undergoing cardiac surgery | LR, GBM, KNN, NN | 56 | SHAP | Internal – 80% training, 20% testing, 5-fold CV, repeated 20 times, bootstrap |
| 110 | Penny-Dimri et al. (2023) | Retrospective cohort study using ANZSCTS database | To predict 30-day mortality | Australia | 153,932 adult patients undergoing cardiac surgery | UAN (Bayesian uncertainty quantification: GVI, PN), XGBoost, Bayesian LR | 41 | Attention heatmap | Both – 5-fold CV repeated 4 times, MIMIC III dataset |
| 111 | Perduca et al. (2024) | Prospective cohort study | To predict changes in platelet count, prothrombin ratio, fibrinogen assay | France | 598 adult patients | Super Learner (135 algorithms: ENR, GAM, RF, SVM, XGBoost, LR) | 16 | Permutation based importance scores | Internal – 80% training, nested 10-fold CV |
| 112 | Petrosyan, Mesana and Sun (2022) | Retrospective cohort study using Cardiocore | To predict AKI of any severity | Canada | 6522 adult patients undergoing cardiac surgery with CPB | Hybrid (RF + LR), LR, enhanced LR | 43 | Feature importance | Internal – 10-fold CV, 500 bootstrap samples |
| 113 | Peyster et al. (2022) | Retrospective cohort study | To predict cardiac allograft vasculopathy | USA | 302 patients undergoing heart transplantation | LR (HistoCAV-Pr, iCAV-Pr) | 680 | NP | Internal – 66% training, 34% hold-out testing, leave-one-out CV |
| 11 | Raghu et al. (2023) | Retrospective cohort study | To predict postoperative mortality | USA | 9283 adults undergoing cardiothoracic surgery | CNN (Inception V4, TL) | NP | Saliency maps | Both – temporal and external cohorts |
| 115 | Rank et al. (2020) | Retrospective cohort study | To predict stage 2-3 AKI | Germany | 13,895 adult patients undergoing cardiothoracic surgery | RNN | 96 | NP | Internal – 5-fold CV |
| 116 | Rogers et al. (2022) | Retrospective cohort study using ACS-NSQIP database | To determine weighted personalized factors contributing to mortality | USA | 1,291 patients undergoing cardiac surgery | GBM, Stacked Ensemble, DNN, RF, LR | 12 | LIME | Internal – 70% training, 30% testing, 10-fold CV |
| 117 | Ryan et al. (2023) | Retrospective cohort study using MIMIC-IV database | To predict AKI of any severity + stage 2-3 AKI | USA | 4,267 patients undergoing cardiac surgery | Ensemble model (tree-based, NN, clustering, LR via AutoGluon) | NP | Permutation feature importance | Internal – 70% training, 30 testing, 10-fold CV |
| 118 | Santos et al. (2024) | Retrospective cohort study | To predict 90 days severe complications post discharge (dialysis, stroke, sudden death, readmission) | Portugal | 5,045 adult patients undergoing open heart surgery | RF, LGBM, SVM, NB, DT | 58 | SHAP | Internal – 5-fold CV |
| 119 | Sarris et al. (2024) | Retrospective cohort study using ECHSA ECCDB | To predict mortality, prolonged ventilation and length of stay | Greece | 172,888 congenital cardiac surgical procedures | OCT | NP | NP | Internal – 70% training, 30% testing |
| 120 | Schuurmans et al. (2025) | Randomized controlled trial | To reduce hypotension severity | Netherlands | 142 elective on-pump CABG with or without valve surgery | HPI (ML algorithm from arterial waveform analysis) | NP | NP | NP |
| 121 | Sen and Seckiner (2021) | Retrospective cohort study | To predict in-hospital mortality | Turkey | 88 patients undergoing CABG | ANN | 37 | NP | Internal – 10-fold CV |
| 122 | Shen et al. (2025) | Retrospective cohort study | To predict complications (acute kidney injury, atrial fibrillation, cardiac arrest, deep vein thrombosis or pulmonary embolism, blood transfusion, and other intraoperative cardiac events) | USA | 6,502 cardiac surgeries | surgVAE, XGBoost, DNN, Multi-task DNN, LR, RF, GBM | 663 | Integrated gradients | Internal – 5-fold CV with stratification for rare outcomes; non-cardiac cohorts used for data augmentation |
| 123 | Sherman et al. (2022) | Retrospective cohort study using STS ACSD | To predict 30-day hospital readmission | USA | 4,924 adult patients undergoing cardiac surgery | RF, XGBoost, SVC, LR | 165 | SHAP | Internal – 75% training, 25% testing, 5-fold CV, bootstrap sampling |
| 124 | Sinha et al. (2024) | Retrospective cohort study using NACSA database | To predict 30-day mortality | UK | 224,318 adult patients undergoing cardiac surgery | XGBoost | 23 | SHAP | Internal – 70% training, 30% validation, 5-fold CV |
| 125 | Sinha et al. (2023) | Retrospective cohort study using NACSA database | To predicting in-hospital mortality | UK | 227,087 adult patients undergoing cardiac surgery | RF, NN, XGBoost, Weighted SVM, LR (retrained) | 18 | Feature importance | Both – 70% training, 30% temporal validation, 5-fold CV |
| 126 | Song et al. (2024) | Retrospective cohort study | To predict AKI of any registry | China | 701 patients undergoing off-pump CABG | LR, GBDT, XGBoost, AdaBoost, RF, SVM, KNN, DT | 80 | Feature importance | Internal – 80% training, 20% testing, 10-fold CV |
| 127 | Song, Frakes and Dasi (2025) | Computational modelling study | To predict postoperative boundary conditions | USA | 1,000 virtual pediatric patients undergoing pediatric cardiac surgery (Fontan) | GAAL (GAN + encoder-decoder CNN framework) | NP | Latent space visualization | Internal – synthetic test dataset |
| 128 | Squiccimarro et al. (2025) | Retrospective cohort study | To evaluate the impact of systemic inflammatory response syndrome on 30-day mortality | Italy | 1,908 patients undergoing elective or urgent cardiac surgery with CPB | RF, LR, SVM | 10 | SHAP | Internal – 70% training, 15% validation, 15% testing, 5-fold CV |
| 129 | Stošić et al. (2025) | Prospective cohort study | To predict postoperative delirium | Serbia | 131 adult patients undergoing elective or urgent cardiac surgery | XGBoost | NP | SHAP | Internal – 80% training, 20% testing, 5-fold CV on training set |
| 130 | Sudri et al. (2024) | Retrospective cohort study | To evaluate the concordance between multidisciplinary heart team recommendations for coronary revascularization and large language models | Israel | 86 patients undergoing percutaneous or surgical revascularization | ChatGPT-3.5, ChatGPT-4, Med-PaLM2 | NP | NP | NP |
| 131 | Sughimoto et al. (2023) | Retrospective cohort study | To predict postoperative lactate levels | Japan | 48 pediatric ICU patients after cardiac surgery | Hypertuned RF, AdaBoost, DT | NP | Feature importance | Internal – 5 and 10-fold CV |
| 132 | Sun et al. (2025) | Retrospective cohort study | To predict intraoperative allogeneic red blood cell transfusion | China | 766 patients undergoing left anterior thoracotomy minimally invasive direct coronary artery bypass | XGBoost, GB, CAT, LR, RF, AdaBoost, SVM, LDA, ExtraTrees, DT | NP | SHAP | Internal – 80% training, 20% testing |
| 133 | Tadege, Tegegne and Dessie (2024b) | Retrospective cohort study | To predict mortality | Ethiopia | 1,520 patients undergoing cardiac surgery | LR, BN, NN, C5, DecisionList, Discriminant, CHAID, QUEST, regression and classification tree | 12 | NP | Internal – 90% training, 10% testing |
| 134 | Tadege, Tegegne and Dessie (2024) | Retrospective cohort study | To predict long-term survival status | Ethiopia | 1,520 patients undergoing cardiac surgery | RSF, GB-Survival, Survival Tree, SVM | 12 | Feature importance | Internal – 10-fold CV |
| 135 | Tang et al. (2025) | Retrospective cohort study | To predict new-onset atrial fibrillation | China | 92 patients undergoing cardiac surgery | RF, SVM, LR, XGBoost, KNN, LDA | 84 | NP | Internal – 5-fold CV |
| 136 | Tang et al. (2023) | Retrospective cohort study | To predict mortality | China | 2,364 patients undergoing CABG | GBT, RF, LR, KNN | NP | NP | Internal – 60% training, 40% testing |
| 137 | Theis et al. (2023) | Retrospective cohort study | To predict survival using CT imaging | Germany | 760 patients undergoing TAVI | CNN, Autoencoder pretraining, CPHM (DL + CPHM) | NP | NP | Internal – 80% training, 20% testing, 5-fold CV on training set |
| 138 | Thongprayoon et al. (2022) | Retrospective cohort study | To predict AKI of any severity | USA | 13,158 adult patients undergoing open heart surgery | DT, RF, XGBoost, ANN, LR, AutoML | 69 | SHAP and LIME | Internal – 70% training, 15% validation, 15% testing, 10-fold CV repeated 5 times |
| 139 | Tong et al. (2024) | Retrospective cohort study | To predicting 4 major adverse postop outcomes (low cardiac output syndrome (LCOS), pneumonia, renal failure, and deep venous thrombosis) | China | 23,000 pediatric patients undergoing congenital cardiac surgery | LGBM, CAT, LR, SVM, RF | 39 | SHAP | Both – 10-fold CV, temporal validation |
| 140 | Topkara et al. (2022) | Retrospective cohort study using INTERMACS registry | To predict myocardial recovery | USA | 20,270 adult patients undergoing durable continuous-flow mechanical circulatory support | Bayesian LR, Linear SVM, GBDT, NN, RF | 28 | NP | Internal – 70% training, 30% testing, 10-fold CV |
| 141 | Triana et al. (2021) | Retrospective cohort study using STS ACSD | To predict post-surgery length of stay | USA | 2,121 adults with elective or urgent, isolated CABG | ANN, RF | 116 | Feature importance | Internal – 80% training, 20% testing |
| 142 | Truong et al. (2021) | Retrospective cohort study | To predict permanent pacemaker implantation after TAVI | USA | 557 patients with sinus rhythm undergoing TAVI | RF, DT, Conditional Inference Trees, SVM, XGBoost, NN | NP | Permutation based importance scores | Internal – 75% training, 25% testing |
| 143 | Tseng et al. (2020) | Retrospective cohort study | To AKI of any severity | Taiwan | 671 patients undergoing cardiac surgery | LR, SVM, RF, XGBoost, ensemble (RF + XGBoost) | 84 | SHAP | Internal – 70% training, 30% validation |
| 144 | Tsushima et al. (2021) | Retrospective cohort study | To predict need for permanent pacemaker implantation | USA | 1,390 patients undergoing TAVI | LR, LWL, SMO, GB | NP | NP | Both – temporal validation |
| 145 | van Erck et al. (2024) | Retrospective cohort study | To evaluate low muscle quality as predictor for mortality | Netherlands | 1,199 patients undergoing TAVI | DL (Quantib body composition software) | NP | NP | NP |
| 146 | Vasileios et al. (2025) | Retrospective cohort study | To predict new-left bundle branch block | Greece | 469 patients undergoing TAVI | XGBoost, SVM, RF, DT, MLP, LR, AdaBoost, TabPFN, GPT-3.5-turbo, GPT-4 | 20 | NP | Internal – 70% training, 30% testing |
| 147 | R. Wang et al. (2025) | Retrospective cohort study | To detect comorbid coronary artery disease | China | 100 patients undergoing both invasive coronary angiography and coronary CT angiography prior to TAVI | DL (U-Net and NN correction for coronary artery segmentation, CT-FFR) | NP | NP | NP |
| 148 | Wang et al. (2024) | Retrospective cohort study | To predict major adverse cardiovascular events | China | 294 patients with ischemic cardiomyopathy and heart failure with preserved ejection fraction undergoing CABG | Lasso | 4 | NP | Both – 60% training, 40% validation, independent site, 10-fold CV |
| 149 | J. Wang et al. (2025) | Retrospective cohort study | To predict 12-month risk of major adverse cardiovascular and cerebrovascular events, encompassing cardiac death, revascularization, myocardial infarction, and stroke | China | 326 patients with severe aortic stenosis and heart failure with persevered ejection fraction undergoing TAVI | DT, KNN, RF, XGBoost, SVM | 8 | SHAP | Both – 60% training, 40% validation, independent site, 10-fold CV |
| 150 | Wang et al. (2022) | Retrospective cohort study | To predict intraoperative red blood cells transfusion | USA | 2,847 patients undergoing cardiothoracic surgery | Gaussian process regression, gaussian process classification | 202 | Feature importance | Both – 5-fold CV, temporal validation |
| 151 | Wayda et al. (2024) | Retrospective cohort study using Donor Heart Study and Scientific Registry of Transplant Recipients database | To identify predictors of donor heart acceptance | USA | 78,078 adult patients undergoing heart transplantation | RF, LR, Lasso | 18 | NP | Both – 50% training, 50% testing, temporal validation |
| 152 | Wisotzkey et al. (2023) | Retrospective cohort study using PHTS database | To predict 1-year allograft loss | USA | 3787 pediatric patients undergoing heart transplantation | ORSF, ARSF, GB, Cox | 15 | SHAP | Internal – 75% training, 25% testing |
| 153 | Woillard et al. (2021) | Retrospective cohort study | To estimate mycophenolic acid exposure | France | 6,884 patients undergoing heart or renal transplantation | XGBoost | 15 | Permutation feature importance | Both – 10-fold CV, independent cohort |
| 154 | Xue et al. (2024) | Prospective cohort study | To predict warfarin blood levels for personalized dosing | China | 246 adult patients undergoing cardiac surgery | RF, GDA, LR, SVM, MLP, KNN, Bagging | 297 | NP | Internal – training/validation split |
| 155 | T. Yang et al. (2024) | Retrospective cohort study | To predict postoperative delirium | China | 367 adult patients undergoing cardiac surgery | RF, SVM, RBFNN, KNN, KRR | 21 | NP | Internal – 10-fold CV for training and testing |
| 156 | Yang et al. (2021) | Retrospective cohort study | To validate a CNN-based arterial pressure-based cardiac output algorithm | South Korea | 2,057 surgical cases | CNN (transfer learning) | NP | NP | Internal – 95% training, 5% testing |
| 157 | Yang et al. (2025) | Retrospective cohort study | To predict prolonged ICU stay | China | 236 adult patients undergoing cardiac surgery and received IABP | XGBoost, LR, LGBM, MLP, SVM, GNB, CNB | 23 | SHAP | Internal – 90% training, 10% validation, 10-fold CV |
| 158 | Yang et al. (2024) | Retrospective cohort study | To predict prolonged intra-aortic balloon pump use | China | 143 adult patients undergoing CABG and received IABP | LR, LGBM, GNB, MLP, KNN, SVM, CNB | 7 | SHAP | Internal – 90% training, 10% validation, 10-fold CV |
| 159 | Yang et al. (2025) | Retrospective cohort study | To predict bleeding risk within 6 months after CABG | China | 561 patients undergoing CABG | XGBoost, RF, LR, MLP, SVC | 10 | SHAP | Both – 70% training, 30% validation, 5-fold Lasso CV, temporal validation |
| 160 | Yerukala Sathipati et al. (2025) | Case-control study | To predict postoperative atrial fibrillation | USA | 15 adult patients undergoing CABG | RF, XGBoost, KNN, SVM | 10 | SHAP | Both – external validation using Gene Expression Omnibus dataset |
| 161 | Yu et al. (2024) | Prospective cohort study | To monitor left ventricular function using transesophageal echocardiography | Norway | 50 adult patients undergoing on-pump cardiac surgery | CNN | NP | NP | NP |
| 162 | Zea-Vera et al. (2022) | Retrospective cohort study using STS ACSD | To predict mortality, major morbidity, high cost, 30-day readmission | USA | 2,086 adult patients undergoing CABG | XGBoost | 80 (preoperative model), 125 (postoperative model) | Feature importance | Internal – 80% training, 20% testing, 5-fold CV |
| 163 | Zea-Vera et al. (2023) | Retrospective cohort study using STS ACSD | To predict operative mortality, major morbidity, mortality, high hospitalization cost | USA | 4,874 adult patients undergoing cardiac surgery | XGBoost, LR | 25 | Feature importance | Internal – 80% training, 20% testing, 10-fold CV |
| 164 | Z. Zeng et al. (2024) | Retrospective cohort study | To predict AKI of any severity | China | 1,110 patients undergoing off-pump CABG | LR, DT, RF, XGBoost, LR | 39 | SHAP | Internal – 70% training, 30% testing, 5-fold CV |
| 165 | Zeng et al. (2021) | Retrospective cohort study | To predict postoperative complications (cardiac, lung, infectious, others) | China | 1964 pediatric patients undergoing congenital cardiac surgery | XGBoost + K-means | 45 | SHAP | Internal – 70% training, 30% testing, 5-fold CV |
| 166 | Zeng et al. (2024) | Retrospective cohort study using CCSR | To predict in-hospital mortality | China | 168,565 adult patients undergoing cardiac surgery | XGBoost, CatBoost, LGBM, RF, NB, SVC, LR | 20 | Feature importance | Both – temporal validation and spatial external validation |
| 167 | Zeng et al. (2023) | Retrospective cohort study using Paediatric Intensive Care database | To predict AKI of any severity in real-time | China | 3,386 pediatric patients undergoing congenital cardiac surgery | Time-aware attention model (DL), LR, RF, LSTM, GRU, Dipole, RETAIN | 83 | SHAP | Internal – 60% training, 15% calibration, 25% validation, 5-fold CV |
| 168 | Zhang et al. (2023) | Retrospective cohort study | To predict unplanned intra-aortic ballon pump assistance, secondary surgery, intraoperative emergency conversion to on-pump CABG, and mortality | China | 506 patients undergoing off-pump CABG | XGBoost, RF, LGBM, CAT, LR, KNN, NB, SVM | 6 | Feature importance | Internal – 80% training, 20% validation, 5-fold CV |
| 169 | Zhang et al. (2024) | Retrospective cohort study | To predict acute respiratory distress syndrome | China | 1,996 adult patients undergoing cardiac surgery with CPB | DT, GBDT, AdaBoost, XGBoost, LGBM, RF, Deep Forest | 126 | SHAP | Both – 70% training, 30% testing, independent cohort |
| 170 | Zhang, Zhou and Cao (2024) | Retrospective cohort study | To predict heart failure | China | 303 patients with severe coronary calcification and met criteria for coronary rotational atherectomy | XGBoost, LGBM, LR, SVM | 7 | SHAP | Internal – 70% training, 30% validation, 10-fold CV |
| 171 | Zhang et al. (2024) | Retrospective cohort study | To predict need for renal replacement therapy | China | 190 adult patients undergoing CABG | GNB, CNB, LGBM, AdaBoost, MLP, KNN, SVM | 39 | SHAP | Internal – 90% training, 10% validation, 10-fold CV |
| 172 | Zhao et al. (2024) | Retrospective cohort study | To predict postoperative delirium | China | 885 patients undergoing cardiac surgery with CPB | ANN, RF, XGBoost, CatBoost | 8 (preoperative model), 5 (postoperative model) | SHAP | Both – 70% training, 30% testing, temporal validation |
| 173 | Zhong et al. (2021) | Retrospective cohort study using MIMIC-III database | To predict major complications (30-days mortality, hemorrhage requiring reoperation, VTE, stroke) | USA | 6,844 patients undergoing cardiac surgery and admitted to ICU | XGBoost, RF, ANN, LR | 35 | Feature importance | Internal – 80% training, 20% testing, 5-fold CV |
| 174 | Zhou et al. (2024) | Retrospective cohort study using STICH trial | To identify patient subgroups with ischemic cardiomyopathy with greatest survival benefit from CABG vs medical therapy | China | 1212 patients with coronary artery disease and left ventricular ejection fraction lower than 35% | Causal Forest (500 trees ensemble) | 18 | NP | NP |
| 175 | Zhou et al. (2021) | Retrospective cohort study | To predict 1-year mortality | China | 381 patients undergoing heart transplantation | RF, GBM, SVM, LR, XGBoost, AdaBoost, ANN | 13 | SHAP | Internal – 70% training, 30% testing, 5-fold CV, bootstrapping |
| 176 | Zhou et al. (2025) | Prospective cohort study | To predict postoperative delirium | China | 103 patients undergoing preoperative functional MRI scan and cardiac valve replacement | RF, LR, Linear SVM | 20 | Feature importance | Internal – nested 10-fold CV |
| 177 | Zsarnoczay et al. (2025) | Retrospective cohort study | To predict all-cause mortality | Germany | 656 patients undergoing coronary CT angiography before TAVI | NN with VAE | NP | NP | NP |
| 178 | Zweck et al. (2021) | Retrospective cohort study | To predict 1-year mortality | Germany | 1,009 patients undergoing TMVR | XGBoost | 6 | Feature importance | Both – 67% training, 33% validation, 5-fold CV, independent cohort |

**Abbreviations:** ACS-NSQIP, American College of Surgeons National Surgical Quality Improvement Program; AI, artificial intelligence; AKI, acute kidney injury; ANN, artificial neural network; ANZCORS, Australia and New Zealand Congenital Outcomes Registry for Surgery; ANZSCTS, Australia New Zealand Society of Cardiac and Thoracic Surgeons; AutoML, automated machine learning; BME, binary-outcome mixed-effects XGBoost; CABG, coronary artery bypass grafting; CAROLA, Cardiothoracic Anaesthesiology Registry; CCSR, Chinese Cardiac Surgery Registry; CLIATHVR, Chinese Low-Intensity Anticoagulant Therapy after Heart Valve Replacement; CNB, Complement Naive Bayes; CNN, convolutional neural networks; CPB, cardiopulmonary bypass; CPHM, Cox proportional hazards model; CV, cross-validation; DL, deep learning; DNN, deep neural network; DT, Decision Tree; ECDB, European Congenital Heart Surgeons Association Congenital Database; ECG, electrocardiogram; ECHSA ECCDB, European Congenital Heart Surgeons Association Congenital Cardiac Database; ENR, elastic net regularization; EuroSMR, European Registry of Transcatheter Repair for Secondary Mitral Regurgitation; FFNN, feedforward neural network; GAM, generalized additive model; GB, Gradient Boosting; GBC, Gradient Boosting Classifier; GBT, Gradient Boosting Trees; GBM, Gradient Boosting Machine; GNB, Gaussian Naïve Bayes; IABP, intra-aortic balloon pump; IE, infective endocarditis; ISHLT, International Society of Heart and Lung Transplant; KNN, K-Nearest Neighbour Classifiers; LDA, linear discriminant analysis; LGBM, Light Gradient Boosting Machine; LMCA, left main coronary artery; L-OCT, Multivariate Logistic Regression with Optimal Classification Trees; LR, Logistic Regression; LSTM, long short-term memory; LUCCK, learning using concave and convex kernels; LWL, locally weighted learner; MELD, Model for end‐stage liver disease; MIMIC-III, Medical Information Mart for Intensive Care; MLP, MultiLayer Perceptron; M-TEER, mitral valve transcatheter edge-to-edge repair; MVS, mitral valve surgery; NACSA, National Adult Cardiac Surgery Audit; NB, Naïve Bayes; NIS/HCUP, National (Nationwide) Inpatient Sample/Healthcare Cost & Utilisation Project; NN, neural network; NP, not provided; NRD, Nationwide Readmissions Database; OCT, Optimal Classification Trees; OptiML, automated Optimised Model; OPTN, Organ Procurement and Transplantation Network; PCI, percutaneous coronary intervention; PHTS, Paediatric Heart Transplant Society; PRN, Partial Response Network; QDA, quadratic discriminant analysis; RF, Random Forest; RNN, recurrent neural network; RP, recursive partitioning; RSF, Random Survival Forest; SAVR, surgical aortic valve replacement; SCAD-SVM, smoothly clipped absolute deviation-penalised Support Vector Machine; SHAP, SHapley Additive exPlanations; SMO, sequential minimal optimisation; SRTR, Scientific Registry of Transplant Recipients; STICH, Surgical Treatment for Ischemic Heart Failure; STS ACSD, The Society of Thoracic Surgeons Adult Cardiac Surgery Database; SVM, Support Vector Machine; TAVI, transcatheter aortic valve implantation; TMVR, transcatheter mitral valve repair; TTE, transthoracic echocardiogram; T-TEER, tricuspid transcatheter edge-to-edge repair; TTVR, transcatheter tricuspid valve repair; UNOS, United Network for Organ Sharing; VAE, variational autoencoder; VTE, venous thromboembolism; XGBoost, eXtreme Gradient Boosting.

**Supplemental Table 2.** Results of Included Studies.

| **#** | **Reference** | **Best machine learning model outcome** | **Traditional risk model** | **Top 3 predictors** |
| --- | --- | --- | --- | --- |
| 1 | Abbasi et al. (2025) | Deep learning model achieved AUC 0.97 (95% CI, 0.97-0.97) in hemorrhage, AUC 0.92 (95% CI, 0.92-0.92) in VTE, AUC 0.92 (95% CI, 0.92-0.93) in stroke. | None | Hemorrhage: units of postoperative red blood cells, new postoperative arrhythmia requiring ICD, postoperative anoxic brain injury.  VTE: postoperative length of stay, new postoperative arrhythmia requiring ICD, postoperative anoxic brain injury.  Stroke: postoperative length of stay, new postoperative arrhythmia requiring ICD, new postoperative arrhythmia not requiring pacemaker. |
| 2 | Abdul Ghffar et al. (2020) | Fusion Ensemble with STS score achieved AUC 0.96 for 30-day cardiovascular mortality, outperforming STS score alone (AUC 0.8). | STS score | NP |
| 3 | Agasthi et al. (2020) | GBM achieved AUC 0.717 (95% CI 0.696–0.737) for 5-year mortality and AUC 0.716 (95% CI 0.696–0.736) for graft failure. | None | Hospital length of stay, recipient and donor age, donor BMI. |
| 4 | Agasthi et al. (2021) | GBM achieved AUC 0.72 (95% CI 0.68–0.77) for 1-year mortality, which outperformed TAVI2-SCORE (AUC 0.56, 95%CI 0.51–0.62) and CoreValve Score (AUC 0.53, 95% CI 0.47–0.59). | TAVI2-SCORE and CoreValve Score | Cardiac power index, hemoglobin, systolic blood pressure. |
| 5 | Alhwiti, Aldrugh and Megahed (2023) | LR achieved AUC 0.817, LGBM achieved AUC 0.825, and GBC achieved AUC 0.824 for in-hospital mortality. | None | Age, fluid and electrolyte disorders, liver disease. |
| 6 | Allou et al. (2023) | TabBERT achieved AUC 0.834 (95% CI: 0.831–0.838), outperforming EuroSCORE II (AUC 0.807; 95% CI, 0.804-0.809) for in-hospital mortality. | EuroSCORE II | Elective surgery, weight of surgical procedure, creatinine clearance. |
| 7 | Arabyarmohammadi et al. (2024) | CARE pipeline achieved AUC = 0.81, sensitivity of 0.68 and specificity of 0.81 for predicting clinically evident rejection. | None | Endocardial stroma solidity, interstitial stroma eccentricity, lymphocyte foci count. |
| 8 | Aranda-Michel et al. (2022) | LR achieved AUC 0.762, sensitivity of 0.78 and specificity of 0.66 on operative mortality prediction when using MELD score. | None | Cancer, peripheral vascular disease, ejection fraction. |
| 9 | Arian et al. (2022) | SCAD-SVM achieved AUC 0.784 (95% CI: 0.64–0.92), sensitivity 0.59, specificity 0.81 for predicting myocardial function improvement. | None | NP |
| 10 | Ayers et al. (2021) | Ensemble ML achieved AUC 0.764 (95% CI: 0.745–0.782), outperforming IHTSA (0.65), DRI (0.56), RSS (0.61) and IMPACT (0.61) in 1-year mortality. | IMPACT, IHTSA, DRI, RSS | Total bilirubin, mechanical ventilation, lack of ventricular assist device at transplant. |
| 11 | Bartkowiak et al. (2024) | Patients with right ventricular length > 77.4mm had 4x higher risk of composite endpoint (death, HF hospitalization, or reintervention. | None | NP |
| 12 | Behnoush et al. (2023) | LR achieved AUC 0.82 (95% CI: 0.78–0.86), specificity of 0.83 and accuracy of 0.82 for 1-year mortality. | None | Total ventilation hours, ejection fraction, triglycerides. |
| 13 | Benedetto et al. (2020) | Retrained LR and RF achieved similar performance, AUC 0.80 (95% CI: 0.77–0.83), AUC 0.80 (95% CI: 0.76–0.83) in in-hospital mortality, compared to EuroSCORE I and II (AUC 0.76, 95% CI: 0.73–0.79 and AUC 0.77, 95% CI: 0.70–0.84). | EuroSCORE I and II | NP |
| 14 | Bertsimas et al. (2021) | OCT achieved AUC 0.86 for mortality prediction, AUC 0.8 for prolonged MVST prediction and AUC 0.82 for prolonged LOS prediction. | None | Procedure type, patient age, patient weight. |
| 15 | Bertsimas et al. (2022) | OCT identified 17% of hospitals as overperformers and 26.4% as underperformers in case-adjusted mortality after congenital heart surgery. | None | NP |
| 16 | Betts et al. (2023) | GBT achieved AUC 0.87 (95% CI: 0.82–0.92) in validation cohort, well-calibrated (O/E=0.97). This is comparable to PRAiS2 and STS models (AUC 0.86). | PRAiS2 and STS risk models | Patient weight, STAT mortality score, age. |
| 17 | Bi et al. (2022) | XGBoost achieved AUC 0.86 (95% CI: 0.83–0.89) for in-hospital mortality, outperforming APACHE-IV and SOFA (AUC 0.74, 95% CI: 0.69–0.79, AUC 0.68, 95% CI: 0.62–0.74 respectively). | APACHE-IV and SOFA scores | Hospital interval, ICU interval, maximum creatinine level. |
| 18 | Bodenhofer et al. (2021) | RF achieved AUC 0.839 and accuracy of 0.96, outperforming EuroSCORE I (AUC 0.704) and a model trained only on the subset of features EuroSCORE uses (AUC 0.745). | EuroSCORE I | Arterial base excess, cholesterol, C-reactive protein, glomerular filtration rate, EuroSCORE. |
| 19 | Boskovski et al. (2020) | De novo damaging variants were linked to worse transplant-free survival (HR 3.51) and longer ventilation times after CHD surgery, especially in patients without extra-cardiac anomalies (HR 11.21). | None | Worse transplant survival: 15q25.2 and 15q11.2. Prolonged time to final extubation: 15q25.2, 22q11.21, and 3p25.2. |
| 20 | Cai et al. (2021) | XGBoost achieved AUC = 0.806. | None | Duration of surgery, patient weight, duration of cardiopulmonary bypass procedure. |
| 21 | Castela Forte et al. (2022) | RNN achieved AUC 0.82 (95% CI, 0.78-0.86), 0.81 (95% CI, 0.77-0.85), and 0.80 (95% CI, 0.75-0.84) for 30-day, 1-year and 5-year mortality. | None | Lactate dehydrogenase, thrombocytes, urea. |
| 22 | Castela Forte et al. (2021) | Super Learner ensemble achieved AUC (all operations): 0.810 (95% CI: 0.798–0.823), AUC 0.846 (95% CI: 0.812–0.880) for solitary mitral valve, AUC 0.838 (95% CI: 0.813–0.864) for solitary aortic valve. | None | Postoperative urea (day 4), patient age, serum creatinine. |
| 23 | Che et al. (2024) | Extreme RF achieved a mean absolute error of 1.17, mean square error of 2.95, root mean square error of 1.72, and an R2 value of 0.50. | None | Erythrocytes, estimated operation duration, body weight. |
| 24 | Chen et al. (2022) | SVM achieved AUC 0.88. | None | Compliance, spontaneous respiratory rate (SRR), SpO₂. |
| 25 | Chen et al. (2024) | LR achieved AUC 0.761 (95% CI: 0.737–0.784). | None | Age, hypertension, diabetes. |
| 26 | Chen et al. (2020) | Cytokine-based LR achieved AUC 0.87 in screening cohort and 0.86 in validation cohort. | None | Interferon-γ (IFN-γ), Interleukin-16 (IL-16), Macrophage inflammatory protein-1 alpha (MIP-1α). |
| 27 | Couto et al. (2024) | Random Forest achieved RMSLE 0.412 (95% CI 0.405–0.419) on training set, 0.454 (95% CI 0.441–0.468) on validation set. | None | Non-elective surgery, admission to public hospital, heart failure. |
| 28 | Cruz et al. (2024) | NB and RF achieved AUC 0.83 ± 0.05 and 0.82 ± 0.04 in mortality, | None | Medicare insurance coverage, comorbid liver disease, congestive heart failure. |
| 29 | Cruz et al. (2024) | XGBoost regression achieved R² = 0.519 for cost prediction; XGBoost classification achieved AUC 0.895 for high-cost prediction. | None | Elective procedure status, prolonged mechanical ventilation, new-onset respiratory failure or myocardial infarction. |
| 30 | da Cunha et al. (2024) | LR achieved AUC 0.735 (95% CI: 0.7203–0.7497), outperforming TRACK (AUC 0.6757, 95% CI: 0.6609 to 0.6906) and TRUST (AUC 0.6622, 95% CI: 0.6473 to 0.6906). | TRACK and TRUST scoring systems | Hemoglobin level, age, body surface area. |
| 31 | da Cruz et al. (2021) | RF achieved AUC 0.842 ± 0.014. | None | Fluid electrolyte imbalance, Elixhauser score, valvular disease. |
| 32 | Dai et al. (2023) | XGBoost achieved AUC 0.80 (95% CI: 0.683–0.917) and sensitivity of 0.717. | None | Intraoperative urine output, cumulative time of mean arterial pressure lower than 65 mmHg outside cardiopulmonary bypass and autologous blood transfusion. |
| 33 | Del Gaizo et al. (2024) | TextCNN + AttnToNum achieved AUC 0.821 (95% CI: 0.806–0.834), TextLSTM + AttnToNum achieved AUC 0.825 (95% CI: 0.809–0.843). | None | High creatinine values, High BUN (blood urea nitrogen) values. |
| 34 | Dong et al. (2025) | XGBoost achieved AUC 0.8513 (95% CI 0.8221-0.8782), exceeding PRECISE-DAPT (0.7427, 95% CI 0.7028-0.7836). | PRECISE-DAPT score | Ejection fraction drop value, international normalized ratio and platelet count. |
| 35 | Dong et al. (2024) | Standardized XGBoost NC model achieved CEM 0.741, AUC 0.854, 95%CI: 0.7405–0.7411. | None | NP |
| 36 | Duong et al. (2022) | DT achieved AUC 0.72 (95% CI: 0.63–0.81), sensitivity of 0.53 (95% CI: 0.40–0.66), specificity of 0.89 (95% CI: 0.67–0.99), PPV of 0.94 (95% CI: 0.81–0.98) on postoperative day 4. | None | Aortic valve replacement or subaortic stenosis repair or Konno procedure, ventricular L-looping and atrioventricular valve replacement. |
| 37 | Elmahrouk et al. (2024) | RF achieved AUC 100%, accuracy 98%, precision 100%, recall 97%, F1 value 98%, outperforming EuroSCORE II (AUC 0.736, OR: 1.13, 95% CI: 1.09–1.18). | EuroSCORE II | Preoperative lactate levels, emergency surgery, CKD |
| 38 | Evertz et al. (2022) | Fully automated LVEF achieved similar performance to manual: AUC 0.686 in CV mortality prediction (manual: AUC 0.661, p=0.21). Automated quantification saved ~10 minutes per patient. | None | NP |
| 39 | Fan et al. (2022) | Deep Forest achieved AUC 0.856 (95%CI 0.813–0.899). | None | NT-proBNP, heart-type fatty acid binding protein (H-FABP), and uric acid. |
| 40 | Fan et al. (2023) | RF achieved AUC 0.87, sensitivity 0.83, specificity 0.92, accuracy 0.89. Outperformed EuroSCORE I (AUC 0.70), EuroSCORE II (AUC 0.73), STS score (AUC 0.71), and LR (AUC 0.74). | EuroSCORE I, II, STS score | Age, Left Ventricular Ejection Fraction (LVEF), chronic lung disease. |
| 41 | Fernandes et al. (2021) | XGBoost model (outside CPB phase) achieved AUC 0.88 (95% CI 0.83–0.94), specificity 0.85, and sensitivity 0.75. | None | NP |
| 42 | Fliegenschmidt et al. (2022) | Postoperative AI screening achieved AUROC 0.79 (95% CI: 0.69–0.87), Sensitivity 0.74, Specificity 0.63, NPV 0.89. | Traditional risk assessment | Open heart surgery, postinterventional hospitalization, length of ICU stay. |
| 43 | Fragasso et al. (2023) | RF achieved AUC 0.93 (95% CI 0.92–0.94) for binary AKI (AKI of any severity of no AKI), and AUC 0.99 (95% CI 0.98–1) for severe AKI (no-mild or severe AKI). | None | Creatinine, platelets, adrenaline support. |
| 44 | Gao et al. (2023) | LR achieved AUC 0.873 (95% CI, 0.796–0.950), sensitivity of 0.80, specificity of 0.87, accuracy of 0.84, PPV of 0.80, and NPV of 0.87. | None | Antithrombin activity, platelet count, fibrinogen level. |
| 45 | Giangreco et al. (2021) | LR using KLKB1 and inotrope therapy panel achieved AUC 0.7181 (95% CI: 0.7020–0.7372), and AUROC of 0.71 in validation cohort. This outperforms the radial score, MEED score, and CVP/PCWP ratio by 50% on average. | Radial score, MEED score, CVP/PCWP ratio | KLKB1 (plasma kallikrein), PRDX2 (peroxiredoxin 2), tropomyosin alpha-4 (TPM4) . |
| 46 | Gomes et al. (2021) | RF achieved AUC 0.97 for all-cause in-hospital mortality prediction, outperforming STS risk score (AUC 0.64), the STS/ACC TAVR score (AUC 0.65). | STS risk scores and STS/ACC TAVR risk score | Baseline Troponin T, Peak Leukocyte Count, Septum Thickness (Echo). |
| 47 | Griffeth et al. (2024) | XGBoost model achieved AUC 0.782 (95% CI 0.568 – 0.804) in validation cohort. | None | Systemic ventricular ejection fraction, serum creatinine, age. |
| 48 | Habertheuer et al. (2021) | STOP score achieved AUC 94.0% for operative morbidity prediction and 94.1% for operative mortality prediction. | None | Mortality: dialysis, active endocarditis, lung disease.  Morbidity: dialysis, emergent intervention, multivalve procedure. |
| 49 | Halloran et al. (2024) | MMDx system identified 7 rejection archetypes, with 48% showing no rejection and reduced graft survival linked to TCMR, severe-recent injury, and atrophy-fibrosis—even without rejection. | None | NP |
| 50 | Haregu et al. (2025) | CatBoost achieved AUC 0.74 (95% CI 0.69–0.79), recall 0.75. | None | Candidate diagnosis, age/size, ventilator use. |
| 51 | Hausleiter et al. (2024) | EuroSMR XGBoost model achieved AUC 0.789 (95% CI: 0.737–0.842) in the validation cohort, outperforming EuroSCORE II, MitraScore and COAPT risk score. | EuroSCORE II, MitraScore, COAPT risk score | NT-proBNP, NYHA class, hemoglobin. |
| 52 | Hui et al. (2023) | Ensemble Voting Classifier achieved AUC 0.738 using the Papworth definition of bleeding, outperforming Papworth Bleeding Risk Score (AUC 0.471). | Papworth Bleeding Risk Score | Hemoglobin in ICU, noradrenaline dose, serum albumin in ICU. |
| 53 | Hur, Wi and Kim (2020) | LSTM with attention achieved AUC = 0.97, F1 score = 0.82. | None | NP |
| 54 | Jia et al. (2023) | LGBM achieved AUC 0.80 (95% CI: 0.75-0.85). | None | Serum creatinine in first 24hr after surgery, last preoperative sCr level, body surface area. |
| 55 | Jiang et al. (2025) | XGBoost achieved AUC 0.793 ± 0.012, mean precision 0.700 ± 0.060, Brier score 0.150 ± 0.023. | None | Mean anion gap (24 h pre-extubation), creatinine level, positive end-expiratory pressure (PEEP). |
| 56 | Jiang et al. (2023) | LR performed best for PO-AKI (AUC 0.812, 95% CI: 0.75-0.86), sensitivity 0.774, accuracy 0.753. GBC performed best for severe AKI (AUC 0.86, 95% CI: 0.81-0.90) | None | Preoperative serum creatinine, hyperlipidemia, lipid-lowering agents for both postoperative AKI and severe AKI. |
| 57 | Kachroo et al. (2022) | LR achieved AUC 0.75, accuracy 0.66, sensitivity 0.71, specificity 0.65. | None | Bypass graft to the ramus intermedius, second obtuse marginal, and second diagonal coronary artery branches, peripheral vascular disease. |
| 58 | Kageyama et al. (2024) | LASSO regression + Cox model achieved AUC 0.935, C-index 0.924, outperforming SS2020. | SYNTAX Score II 2020 | Pulmonary hypertension, COPD, insulin-dependent diabetes. |
| 59 | Kainuma et al. (2022) | RSF identified predictors of 1-year mortality: liver function in early re-transplant group and obesity and preoperative ventilator dependence in late re-transplantation. | None | Early re-transplant group: total bilirubin >2 mg/dl. Late re-transplant group: high BMI >30 kg/m², ventilator dependence. |
| 60 | Kalisnik et al. (2022) | XGBoost achieved AUC 0.88, sensitivity 0.78, specificity 0.78, accuracy 0.82. | None | eGFR at 6–12h postoperatively, change in eGFR at 6/12h postoperatively, age. |
| 61 | Kampaktsis et al. (2023) | CatBoost achieved AUC 0.80 (95% CI: 0.687–0.811) and accuracy of 0.75 for 1-year mortality prediction. In the 3-year model, it achieved AUC 0.69, accuracy 0.74. | None | Post-transplant hemodialysis, eGFR, ischemic time. |
| 62 | Kampaktsis et al. (2021) | AdaBoost achieved AUC 0.689 (95% CI: 0.665–0.715), sensitivity 0.63, specificity 0.69, PPV 0.22, NPV 0.94 in 1-year mortality prediction, outperforming IMPACT score (AUC 0.569, 95% CI .545–.592). It achieved AUC 0.629 (95% CI .617–.638) in 1-year acute rejection prediction. | IMPACT score | Recipient serum creatinine, recipient height, ischemic time. |
| 63 | Karri et al. (2021) | GBM achieved AUC 0.74 (95% CI: 0.71–0.77), sensitivity 73%, specificity 64%, outperforming POAF score (AUC 0.63, 95% CI: 0.62–0.64). | POAF Score | Age, coagulopathy, valvular disease. |
| 64 | Kilic, Habib, et al. (2021) | Predictive performance was markedly better in patients with concordant XGBoost and STS risk estimates in all outcomes except deep sternal wound infection. | None | Concordance: diabetes mellitus, hypertension, serum creatinine.  Discordance: age, female sex, white race. |
| 65 | Kilic, Goyal, et al. (2021) | XGBoost achieved higher c-index for five of seven outcomes: operative mortality: 0.771 vs 0.762 (STS), prolonged ventilation: 0.739 vs 0.726, acute renal failure: 0.776 vs 0.737, reoperation: 0.637 vs 0.626, composite outcome: 0.703 vs 0.690. | STS risk model | Age, chronic lung disease, serum creatinine. |
| 66 | Kim et al. (2022) | RF achieved AUC 0.94 on cardiac surgery cohort (training), 0.94 on vascular surgery (testing), and 0.82 on non-cardiac cohort (testing) for 0.5-hour prediction window. | None | Platelet count, intubation status, creatinine. |
| 67 | Kim and Jang (2021) | RF achieved AUC 0.87, sensitivity 0.81, specificity 0.88, accuracy 0.86. | None | Steroid use, labile INR, history of stroke. |
| 68 | Kirchner et al. (2024) | AI-based CT RVEF <50% and RVEDV >210 ml predicted combined endpoint (death or HF rehospitalization) with HR 2.864 and 3.753 respectively. | None | LVEF, gender, EuroSCORE II. |
| 69 | Kobayashi et al. (2023) | RNN achieved MAE 1.96 mmol/L (IQR 1.87–2.05). | None | Intraoperative lactate concentration, baseline anemia, patient weight. |
| 70 | Kurmanaliyev et al. (2025) | RF achieved accuracy 0.8571, precision 0.9, recall 0.7826, F1-score 0.8372. | None | Left femoral artery diameter, aortic valve calcification volume, angle of aortic annulus. |
| 71 | Kwak et al. (2021) | RSF achieved C-index: 0.778 in 3-year mortality prediction. | None | Atrial fibrillation, extracellular volume fraction percentage and right ventricular ejection fraction. |
| 72 | Lachmann et al. (2022) | DL model achieved AUC 0.687 (95% CI: 0.642–0.731) for predicting 1-year mortality, outperforming TAPSE (AUC 0.625, P=0.029). DL also outperformed MitraScore, achieving AUC 0.687 (95% CI, 0.642–0.731), versus 0.662 (95% CI, 0.618–0.706), though not statistically significant p = 0.284. | Tricuspid annular plane systolic excursion (TAPSE), MitraScore | NP |
| 73 | Lachmann et al. (2025) | AI-based phenotyping identified high-risk clusters with persistent right heart dysfunction and reduced 2-year survival (69.2% and 74.6%) compared to low-risk clusters (88.3% and 85.5%) after TAVR. | None | NP |
| 74 | Li et al. (2023) | XGBoost achieved AUC 0.913 (95% CI: 0.910–0.916), accuracy 0.86, sensitivity 0.85, specificity 0.86, F1 score 0.294. Model performance on the selected variable set was better than that on GRACE variables. | GRACE score | Heart rate, elevated creatinine, left atrial diameter. |
| 75 | Li et al. (2023) | CatBoostClassifier achieved AUC 0.85 and 0.67 in testing and external. | None | NT-proBNP, BUN, prothrombine time. |
| 76 | Li et al. (2025) | XGBoost achieved the highest AUC 0.82 ± 0.08. | None | Surgical methods, age, number of interval differences of successive normal heart beats greater than 50 ms. |
| 77 | Li et al. (2024) | RF achieved AUC 0.92 (95% CI: 0.91–0.92) with full feature set and AUC 0.86 (95% CI: 0.85–0.88) with selected feature set. | None | Pain score, anesthesia duration, pH level. |
| 78 | Li et al. (2022) | LR achieved AUC 0.821 (95% CI: 0.740–0.901) vs AUC: 0.654 (95% CI: 0.545–0.763) of Cleveland Clinic score, with higher sensitivity, specificity and accuracy. | Cleveland Clinic score | Serum cystatin C, eGFR, right atrial long-axis dimension. |
| 79 | Li et al. (2024) | CatBoost achieved highest AUC 0.68 (testing) and 0.67 (external validation) at 100x and 70x URL cut-offs respectively. | None | 40x URL: high-sensitivity CRP (Hs-CRP), CABG surgery type, neutrophil count.  70x URL: CPB time, NT-pro BNP, CABG surgery.  100x URL: CPB time, aortic time, surgery time.  130x URL: CPB time, aortic time, surgery time. |
| 80 | Li et al. (2020) | NB achieved AUC 0.755 and 0.845 in AKI and severe AKI. | None | Gender, LVEF, serum creatinine. |
| 81 | Li et al. (2025) | RF achieved AUC 0.91 (95% CI: 0.89–0.93), accuracy 95%, precision 97%, recall 53%, F1-score 0.55 on test dataset. | None | Moderate to severe MR, mitral valve leaflet thickening, ejection fraction. |
| 82 | Liou et al. (2025) | Random Survival Forests and Cox Gradient Boost had the highest performances with C-indices of 0.628 and 0.627. | None | Age, total ischemic time, bilirubin. |
| 83 | Lisboa et al. (2022) | PRN-Lasso achieved AUC 0.628 (95% CI: 0.602–0.654), comparable to IHTSA (AUC 0.635, 95% CI: 0.609–0.662), and outperforming IMPACT (AUC 0.602, 95% CI: 0.575–0.628). | IMPACT | Donor age, ischemic time, creatinine, ventilator at OHT, transplant year. |
| 84 | Liu et al. (2024) | FC + self-attention achieved MAE 0.9388 (test), 1.1325 (validation), MAPE 0.1821 (test), MSE 1.6821 (test). | None | Gap to target aPTT, thrombin time, history of coronary heart disease |
| 85 | Liu et al. (2022) | Personalized model with transfer learning outperformed the global model for AKI estimation among general inpatients (AUC 0.78, 95% CI, 0.77-0.79 vs 0.76, 95% CI, 0.75-0.76) and across the high-risk subgroups (0.79, 95% CI, 0.78-0.80 vs 0.75, 95% CI, 0.74-0.77) and low-risk subgroups (0.74, 95% CI 0.73-0.75 vs 0.71, 95% CI 0.70-0.72). | None | NP |
| 86 | Lu et al. (2023) | SVM achieved AUC 0.777, Accuracy 0.72, Specificity 0.80, PPV 0.63, Sensitivity 0.60, NPV 0.77. | None | Left atrial diameter, postoperative WBC count, age. |
| 87 | Luo et al. (2022) | XGBoost (SYSUPMIE model) achieved AUC 0.813 (95% CI: 0.670–0.933), outperforming EuroSCORE II (0.632, 95% CI: 0.479–0.761) | EuroSCORE II | Serum albumin, platelet count, current heart failure. |
| 88 | Luo et al. (2023a) | XGBoost achieved 0.890 (95% CI 0.876-0.906), 0.912 (95% CI 0.899-0.924) with intraoperative variables. | None | Serum creatinine, perfusion time, body length. |
| 89 | Ma et al. (2021) | Stratified sampling NN improved low-dose prediction (external validation: ideal prediction increased from 0.7% to 9.6%) while maintaining overall accuracy (76.4% vs. 75.6%). | None | NP |
| 90 | Mao et al. (2025) | RF achieved AUC 0.793 (95% CI: 0.712–0.873) in derivation cohort and consistent performance in external validation. | None | NP |
| 91 | Marmagkiolis et al. (2024) | Active cancer reduced odds of receiving TMVR/TEER (OR 0.28; BAM-PS model); no increase in mortality or cost observed. | None | NP |
| 92 | Mathis et al. (2022) | RF achieved AUC 0.803 (95% CI: 0.799–0.807) on training/validation set and AUC 0.709 (95% CI: 0.705–0.712) on temporal holdout test set. | None | NP |
| 93 | Mohammadyari et al. (2024) | DeepSurv achieved C-index 0.835. | None | Albumin, Age, Body Mass Index. |
| 94 | Molina et al. (2022) | GBM achieved AUC 0.755 (95% CI: 0.666–0.838), outperforming EuroSCORE II (AUC 0.716, 95% CI: 0.630-0.800). | EuroSCORE II | Chronic obstructive pulmonary disease, tricuspid regurgitation, and urgency upon admission. |
| 95 | Mori et al. (2021a) | XGBoost with pre and intraoperative variables achieved best AUC compared to using pre or intraoperative variables alone | None | Plasma transfusion, any transfusion, intraoperative IABP use. |
| 96 | Mustafa et al. (2025) | ANN achieved AUC 0.80, F1 score 0.87, Accuracy 80%; XGBoost achieved similar AUC 0.80, F1 score 0.86. | None | Patient disposition, elective procedure status, coagulable disorders |
| 97 | Nagy et al. (2024) | AUC 0.88 (95% CI: 0.72 - 1.00), precision of 0.92 (95% CI: 0.70–1.00), accuracy of 0.91 (95% CI: 0.82–1.00). | None | Preoperative serum creatinine, surgery duration, POD0 serum pH. |
| 98 | Namasivayam et al. (2022) | Ridge regression achieved AUC 0.74 (primary cohort), AUC 0.78 (validation cohort) for combined outcome (death/AVR). | None | NP |
| 99 | Narayan et al. (2022) | LR achieved AUC 0.705, specificity 0.965, sensitivity 0.158. | None | Age, body mass index, hypertension. |
| 100 | Nistal-Nuño (2022) | XGBoost achieved AUC 0.926, Brier Score 0.041, accuracy 0.88, sensitivity 0.80, specificity 0.88, outperforming OASIS, SAPS II, SAPS III. | OASIS, SAPS II, SAPS III | Age, ICU admission type (elective/urgent/emergency), and CABG surgery presence. |
| 101 | Nowakowska et al. (2024) | RF achieved AUC 0.671, sensitivity 0.701 and specificity 0.528 in depression. GBT achieved AUC 0.781, sensitivity 0.719, specificity 0.723 in delirium. | None | Depression: sRAGE, Clock Drawing Test (CDT) score, diabetes. Delirium: age, MCP-1, antioxidant activity. |
| 102 | Okuno et al. (2021) | Autoencoder achieved AUC 0.79 (95% CI: 0.65–0.93) for predicting 30-day CVE. | None | NP |
| 103 | Orfanoudaki et al. (2022a) | XGBoost achieved AUC 0.826, LR AUC 0.825 (STS AUC: 0.796) in mortality, XGBoost achieved AUC 0.819 (STS: 0.788) in prolonged ventilation, XGBoost AUC 0.815 (STS: 0.7909) in renal failure. | STS risk models | NP |
| 104 | El Ouahidi et al. (2024) | SVM achieved AUC 92.1% ± 4.7, F1 Score 71.8% ± 9.9, Accuracy 87.9% ± 4.7, outperforming Emory Risk Score (AUC of 62.3% ± 8.5). | Emory Risk Score | NP |
| 105 | Ouyang et al. (2024) | PreOpNet achieved AUC 0.83 (95% CI: 0.79–0.87), outperforming RCRI score (AUC 0·67, 95% CI: 0.61–0.72). | RCRI score | NP |
| 106 | Pan et al. (2025) | XGBoost achieved AUC 0.96 (95% CI: 0.93–0.98), accuracy 93%, sensitivity 86%, specificity 94%. | None | IL-6 on POD3, procalcitonin on POD3, CXR exudation. |
| 107 | Pei et al. (2025) | NB achieved AUC 0.7936 and 0.7410 on in-hospital and 360-day mortality. | None | Postoperative stress hyperglycemia ratio, male sex, age. |
| 108 | Pekař et al. (2024) | AutoMATiCA-assisted analysis identified SMI, VAT density, and SAT density as significant predictors (e.g., SMI HR: 0.986, VAT density HR: 1.015, SAT density HR: 1.014). | None | NP |
| 109 | Penny-Dimri et al. (2021) | GBM achieved AUC 0.78 (SD: 0.01) in AKI, outperforming the Cleveland Clinic Score AUC 0.75 (SD: 0.004). | Cleveland Clinic Score | Total CPB time, preoperative creatinine, age. |
| 110 | Penny-Dimri et al. (2023) | UAN-GVI achieved AUC 0.78 on external validation; improved to AUC 0.82 for high-confidence predictions. | None | Age, BMI, preoperative creatinine. |
| 111 | Perduca et al. (2024) | Super Learner achieved RMSE: platelet count 38.27×10⁹/L, prothrombin ratio 8.66%, fibrinogen assay 0.44 g/L. | None | Sex, weight, body mass index. |
| 112 | Petrosyan, Mesana and Sun (2022) | Hybrid ML achieved AUC 0.75, sensitivity 0.67, specificity 0.94, PPV 0.50, NPV 0.88. | None | CARE score 4, preoperative eGFR, CARE score 3. |
| 113 | Peyster et al. (2022) | iCAV-Pr (combined model) achieved best performance: AUC 0.933, accuracy 0.89 on the test set. | None | NP |
| 11 | Raghu et al. (2023) | CXR-CTSurgery showed similar discrimination to STS-PROM (AUC 0.83 vs 0.88; 0.74 vs 0.80) with improved calibration (O/E ratio: 0.74 vs 0.52; 0.91 vs 0.73). | STS Predicted Risk of Mortality (STS-PROM) | NP |
| 115 | Rank et al. (2020) | RNN achieved AUC 0.893 (95% CI: 0.862–0.924), outperforming physicians (AUC 0.901 vs. 0.745, p < 0.001). | None | NP |
| 116 | Rogers et al. (2022) | GBM achieved AUC 0.637 (95% CI: 0.559–0.715). | None | Preoperative dialysis, emergent cases, Hispanic ethnicity. |
| 117 | Ryan et al. (2023) | Dynamic model achieved AUC 0.95 for Stage 2+ AKI prediction within 48 hours. Static model achieved AUC 0.87 (Stage 2+ AKI, first 72 hours). | None | Maximum creatinine (dynamic), baseline creatinine (static), minimum cardiac output (dynamic) |
| 118 | Santos et al. (2024) | RF achieved AUC 0.717 (training), 0.695 (testing), 0.653 (testing). | None | Number of in-hospital complications, ICU stay duration, surgery duration. |
| 119 | Sarris et al. (2024) | OCT achieved AUC 0.866 for mortality, 0.851 for prolonged ventilation, 0.818 for length of stay. | None | NP |
| 120 | Schuurmans et al. (2025) | HPI-guided care reduced time-weighted average of hypotension by 63% (median difference –0.40 mm Hg, 95% CI –0.65 to –0.27; p<0.001) and time spent in hypotension by 28 minutes (95% CI 17–44 min). | None | NP |
| 121 | Sen and Seckiner (2021) | ANN using batch back propagation algorithm achieved correct classification rate 99.6% and mean network error 0.05. | None | NP |
| 122 | Shen et al. (2025) | surgVAE achieved macro-averaged AUC 0.831 and AUPRC 0.409. | None | Atrial fibrillation: preoperative AF condition, age, and platelet count.  Blood transfusions: hematocrit, hemoglobin, and platelet count.  Intraoperative cardiac events: NT-proBNP level, platelet count, and red cell distribution width. |
| 123 | Sherman et al. (2022) | RF achieved AUC 0.76 (95% CI: 0.73–0.79), Brier score 0.16 (95% CI: 0.15–0.17). | RF using STS variables only | Length of hospital stay, discharge to rehabilitation or home, ICU length of stay. |
| 124 | Sinha et al. (2024) | XGBoost achieved AUC 0.846 (95% CI: 0.845–0.846), F1 0.277. | None | Surgery type, age, creatinine clearance. |
| 125 | Sinha et al. (2023) | XGBoost achieved AUC 0.834 (95% CI: 0.834–0.834), F1-score 0.278, RF achieved similar AUC (0.834, 95% CI: 0.833–0.834). Both outperformed EuroSCORE II (AUC 0.818, 95% CI: 0.817–0.818). | EuroSCORE II | Operative urgency, age, New York Heart Association (NYHA) score. |
| 126 | Song et al. (2024) | GBDT achieved AUC 0.739, accuracy: 0.943. | None | Use of insulin aspart, use of acarbose, use of spironolactone. |
| 127 | Song, Frakes and Dasi (2025) | Diversity-aware GAAL achieved prediction accuracy 90.41% and R² 0.88. | None | NP |
| 128 | Squiccimarro et al. (2025) | RF model achieved AUC 0.74 (95% CI 0.69-0.80). | None | NP |
| 129 | Stošić et al. (2025) | XGBoost achieved AUC 0.998, accuracy 97.6%, precision 1.00, recall (sensitivity) 0.95, F1-score 0.976. | None | Sedation with mechanical ventilation, fibrinogen levels. |
| 130 | Sudri et al. (2024) | ChatGPT-4 with detailed input format achieved accuracy 0.82, sensitivity 0.80, specificity 0.83. | None | NP |
| 131 | Sughimoto et al. (2023) | Hypertuned RF achieved MAE 3.38 mg/dL with adaptive model. | None | Previous lactate level, hours after admission, area under arterial waveform curve. |
| 132 | Sun et al. (2025) | XGBoost achieved AUC 0.726 and accuracy 0.854 in the test set. | None | Preoperative hemoglobin <120 g/L, prothrombin time >13.75s, BMI <22.7kg/m². |
| 133 | Tadege, Tegegne and Dessie (2024b) | LR achieved AUC 0.961, Lift value 3.33. | None | Age, saturated oxygen (SPO2), ejection fraction. |
| 134 | Tadege, Tegegne and Dessie (2024) | RSF achieved mean cross-validation accuracy 0.82 and test set accuracy 0.89. | None | Saturated oxygen (SPO2), age, waiting time to surgery, creatinine value. |
| 135 | Tang et al. (2025) | Ensemble ML: achieved accuracy 0.82, sensitivity 0.82, specificity 0.81. | None | Age, left atrium diameter, eGFR. |
| 136 | Tang et al. (2023) | GBT achieved AUC 0.94, F1-score 0.89, and precision 0.87. | None | NP |
| 137 | Theis et al. (2023) | Combined DL+CPHM achieved C-index 0.668, AUC 0.713 in 1-year survival, AUC 0.696 in 2-year survival, outperforming both EuroSCORE I and II. | EuroSCORE I, II | FMF, SMRD, SMA. |
| 138 | Thongprayoon et al. (2022) | AutoML achieved AUC 0.79 (95% CI: 0.77–0.81), RF achieved comparable results AUC 0.78 (95%CI: 0.76–0.80). | None | Baseline eGFR, surgery type, coagulopathy. |
| 139 | Tong et al. (2024) | LGBM achieved AUC 0.893 (95% CI: 0.884–0.895) in low cardiac output syndrome, AUC 0.963 (95% CI: 0.947–0.979) in renal failure, AUC 0.970 (95% CI: 0.953–0.982) in deep vein thrombosis.  LR achieved best performance for pneumonia (AUC 0.929, 95% CI: 0.926–0.931). | None | Low cardiac output syndrome: mechanical ventilation time, anesthesia time, operative time.  Pneumonia: mechanical ventilation time, height, operative time.  Renal failure: mechanical ventilation time, STAT score, operative time.  Deep vein thrombosis: mechanical ventilation time, anesthesia time, operative time. |
| 140 | Topkara et al. (2022) | Bayesian LR achieved AUC 0.824, sensitivity 0.727, specificity 0.790, outperforming INTERMACS recovery risk score (AUC 0.796). | INTERMACS recovery risk scores | Bridge-to-recovery implant strategy, current tobacco abuse, postpartum cardiomyopathy. |
| 141 | Triana et al. (2021) | ANN with top 10 variables achieved MAE 1.612 days, R² = 0.15 in prospective validation. | None | Intubation duration, last preoperative creatinine, age. |
| 142 | Truong et al. (2021) | RF using post-TAVI ECG data achieved AUC 0.81, accuracy 0.76, outperforming RF model without post-TAVI ECG data (AUC 0.72, accuracy 0.70). | None | Delta QRS, delta PR, baseline QRS interval. |
| 143 | Tseng et al. (2020) | Ensemble model (RF + XGBoost) achieved AUC 0.843 (95% CI 0.778–0.899). | None | Intraoperative urine output, units of packed red blood cells transfused during surgery, and preoperative hemoglobin level. |
| 144 | Tsushima et al. (2021) | SLR and LWL achieved highest AUC 0.82 in validation cohort and accuracy up to 75%. | None | Preprocedural right bundle branch block, atrioventricular block. |
| 145 | van Erck et al. (2024) | DL model identified low muscle quality is associated with higher mortality HR 1.40 (95% CI: 1.15–1.70). | None | NP |
| 146 | Vasileios et al. (2025) | XGBoost achieved accuracy 0.85, precision 0.79, F1-score 0.82. | None | NP |
| 147 | R. Wang et al. (2025) | DL-based CT-FFR achieved AUC 0.86, sensitivity 0.86, specificity 0.66, accuracy 0.75. | None | NP |
| 148 | Wang et al. (2024) | Lasso achieved C index 0.768 (95% CI, 0.701–0.835). | None | Diabetes, hypertension, the systemic immune‐inflammation index, and NT‐proBNP. |
| 149 | J. Wang et al. (2025) | SVM achieved AUC 0.756 (95% CI 0.631–0.881) in independent validation set with accuracy 85.4%, Brier score 0.119. | None | Triglyceride glucose, NT-proBNP, age. |
| 150 | Wang et al. (2022) | Gaussian Process classification achieved AUC 0.826 for predicting 4+ RBC units transfused, RMSE 0.117 for 0 units and 1.705 for 1–3 units. | None | Veno-arterial extracorporeal membrane oxygenation (ECMO) initiation, ECMO continuous, ECMO cannulation. |
| 151 | Wayda et al. (2024) | RF achieved AUC 0.908 (95% CI, 0.903–0.914) and accuracy 0.83. | None | Older age, hepatitis C infection, LV dysfunction. |
| 152 | Wisotzkey et al. (2023) | Oblique RSF achieved AUC 0.745 (95% CI: 72.2–76.4) with calibration (ICI=2.6%). | None | Congenital heart disease, mechanical circulatory support at transplant, single ventricle anatomy. |
| 153 | Woillard et al. (2021) | XGBoost with 3 samples achieved relative RMSE = 13.2%, R² = 0.914, MPE = 1.4%. | None | NP |
| 154 | Xue et al. (2024) | RF achieved validation correlation coefficient 0.595, MAE 0.362, ideal prediction proportion 59.0%; GDA achieved TPR 95.6% for INR classification. | None | NP |
| 155 | T. Yang et al. (2024) | RF achieved AUC 0.9202, accuracy 0.88, sensitivity 0.69, specificity 0.95, MCC 70.00%. | None | NP |
| 156 | Yang et al. (2021) | CNN model achieved absolute errors of SV were 14.5 (SD 13.4) mL, significantly smaller than commercial APCO device FloTrac. | None | NP |
| 157 | Yang et al. (2025) | XGBoost achieved AUC 0.92 in training, 0.73 in validation, accuracy 0.66, sensitivity 0.70, specificity 0.76. | None | Tracheotomy, albumin, Sv1. |
| 158 | Yang et al. (2024) | Logistic Regression achieved AUC 0.799 (95%CI: 0.711–0.887) and AUC 0.774 (95%CI: 0.630–0.919) in validation. | None | Left ventricular ejection fraction, left ventricular fraction shortening, preoperative intra-aortic balloon pump. |
| 159 | Yang et al. (2025) | XGBoost achieved AUC 0.926 in temporal external validation cohort, showing no difference in AUROC but higher AUPRC compared to PRECISE-DAPT score. | PRECISE-DAPT score | Preoperative eGFR, age, history of surgery. |
| 160 | Yerukala Sathipati et al. (2025) | XGBoost achieved AUC 0.83, accuracy 0.73, sensitivity 0.75, specificity 0.71 on test dataset. | None | hsa-miR-96-5p, hsa-miR-184, hsa-miR-17-3p, and hsa-miR-200-3p. |
| 161 | Yu et al. (2024) | AutoMAPSE achieved lower measurement variability (LSC 2.4 mm hands-free vs 2.9 mm manual) and high concordance rate (97%) for trending LV changes. | None | NP |
| 162 | Zea-Vera et al. (2022) | XGBoost model achieved AUC 0.77, accuracy 0.98, F1 score 0.244, AUC-PR 0.16 in operative mortality, similar to STS-PROMM.  XGBoost model achieved AUC 0.69, accuracy 0.75, F1 score 0.42, AUC-PR 0.33 in major morbidity or mortality, outperforming STS-PROMM. XGBoost achieved AUC 0.75, accuracy 0.77, F1 score 0.52, AUC-PR 0.51 in high cost. XGBoost achieved AUC 0.73, accuracy 0.70, F1 score 0.49, AUC-PR 0.47 in 30-day readmission. Postoperative ML models for major morbidity or mortality and for high cost performed superior to preoperative ML models. Addition of intraoperative parameters further improved machine learning model performance for major morbidity or mortality. | STS risk models | Operative mortality: white blood cell count, platelets, weight.  Major morbidity or mortality: preoperative creatinine, hematocrit, hemoglobin A1c.  High cost: ejection fraction, preoperative creatinine, platelets.  30-day readmission: weight, platelets, creatinine. |
| 163 | Zea-Vera et al. (2023) | XGBoost achieved accuracy 0.95, AUC-PR 0.21, F1 score 0.31 in operative mortality; accuracy 0.71, F1 0.47, AUC-PR 0.47 in major morbidity or mortality; accuracy 0.84, F1 0.62, AUC-PR 0.65 in high cost. It performed similar to STS risk models. | STS risk models | Operative mortality: weight, creatinine, platelets.  Major morbidity or mortality: weight, creatinine, hematocrit.  High cost: hematocrit, creatinine, platelets. |
| 164 | Z. Zeng et al. (2024) | LGBM achieved AUC 0.797 (95% CI: 0.779–0.815) in temporal external validation. XGBoost achieved AUC 0.732 (95% CI: 0.710–0.754) in spatial external validation. They provided only marginal improvements over LR, but still outperformed EuroSCORE II and SinoSCORE II. | EuroSCORE II, SinoSCORE II | Age, left ventricular ejection fraction, estimated glomerular filtration rate. |
| 165 | Zeng et al. (2021) | XGBoost achieved AUC 0.839 (binary classification), macro-AUC 0.850 (multi-label), recall 0.79. It outperformed RACHS-1, ABC, STS risk models. | RACHS-1, ABC, STS risk models | CPB time, patient weight, surgical time. |
| 166 | Zeng et al. (2024) | RF achieved AUC 0.90 (95% CI: 0.86–0.94), accuracy 0.83, recall 0.74, F1 score 0.78, precision rate 0.80. | None | Intraoperative urine volume, intraoperative sufentanil, intraoperative dexmedetomidine. |
| 167 | Zeng et al. (2023) | DL achieved AUC 0.908 (95% CI, 0.907–0.909), 91% of AKI predicted at 24 hours postoperative. | None | C-reactive protein, autologous blood transfusion, mechanical ventilation time. |
| 168 | Zhang et al. (2023) | XGBoost achieved AUC 0.94, accuracy 0.84, F1-score 0.83. | None | Intraoperative ventricular fibrillation, number of saphenous vein grafts, nerve block, venous oxygen saturation, skin incision-bypass time, and hypertension. |
| 169 | Zhang et al. (2024) | Deep forest achieved AUC of 0.882 and a Brier score of 0.809. | None | COPD, preoperative albumin, central venous pressure. |
| 170 | Zhang, Zhou and Cao (2024) | GNB achieved AUC 0.856 (95% CI: 0.805–0.954) in training and AUC 0.817 (95% CI: 0.630–0.958) in the validation set. | None | Cardiac troponin T (cTnT), creatine kinase isoenzyme (CK-MB), albumin (ALB). |
| 171 | Zhang et al. (2024) | XGBoost achieved AUC 0.972 (95% CI: 0.951–0.994), accuracy 0.92, Sensitivity 1.00, specificity 0.89. | None | Blood cholesterol, serum creatinine, fasting blood glucose, age, triglyceride and NT-proBNP. |
| 172 | Zhao et al. (2024) | ANN achieved AUC 0.929 (preoperative) and 0.925 (postoperative) in test set; validation AUC 0.776 (preoperative) and 0.684 (postoperative). | None | Preoperative: ASA, cerebrovascular disease, education level; Postoperative: APACHE II score, preoperative MMSE score, CPB duration. |
| 173 | Zhong et al. (2021) | XGBoost achieved AUC 0.88, F1 score 0.58 in 30-day mortality, AUC 0.98, F1 score 0.70 in septic shock, AUC 0.88, F1 score 0.55 in thrombocytopenia, AUC 0.89, F1 score 0.40 in liver dysfunction. | None | 30-day mortality: vasopressin, pH, creatinine.  Septic shock: hemoglobin, hematocrit, lactate. Thrombocytopenia: vasopressin, bicarbonate, lactate. Liver dysfunction: PTT, gender, PO₂. |
| 174 | Zhou et al. (2024) | Causal Forest identified. Subgroup with LVESVI >84 mL/m² and age ≤60.27 years had significantly reduced risk of death (aHR 0.61, 95% CI 0.45–0.84, p=0.002) and cardiovascular death (aHR 0.63, 95% CI 0.45–0.89, p=0.009) with CABG. | None | Left ventricular end-systolic volume index, serum creatinine, age |
| 175 | Zhou et al. (2021) | RF achieved AUC 0.801 (95% CI: 0.697–0.891), accuracy 0.83, sensitivity 0.27, specificity 0.93. | None | Albumin, recipient age, left atrium diameter. |
| 176 | Zhou et al. (2025) | RF with functional MRI and clinical variables achieved AUC 0.928, Accuracy 0.88, Sensitivity 0.91. | None | Anterior cingulate, dorsal frontal cortex, praecuneus/posterior cingulate, angular gyrus, med cerebellum, and temporal lobe. |
| 177 | Zsarnoczay et al. (2025) | AI algorithm shown here can efficiently perform fully automated CT-based quantification of LACI, which independently predicts overall mortality after TAVI. | None | Obesity, current smoking, atrial arrhythmia. |
| 178 | Zweck et al. (2021) | XGBoost achieved AUC 0.783 (95% CI: 0.716–0.849), sensitivity 0.70, specificity 0.77. | None | Urea, hemoglobin, NT-proBNP. |

**Abbreviations:** ABC, Age, Biomarkers, Clinical history risk score; AI, artificial intelligence; ANN, Artificial Neural Network; ASA, American Society of Anaesthesiologists; AutoMAPSE, Automated MAPSE Estimation; AutoML, Automated Machine Learning; Cleveland, Cleveland Clinic Score; CNN, Convolutional Neural Network; COAPT, Cardiovascular Outcomes Assessment of the MitraClip Percutaneous Therapy; COPD, chronic obstructive pulmonary disease; CPB, cardiopulmonary bypass; CPHM, Cox Proportional Hazards Model; CVP/PCWP, Central Venous Pressure / Pulmonary Capillary Wedge Pressure; DAPT, Dual Antiplatelet Therapy; Deep Forest, Deep Forest; DeepSurv, Deep Survival Model; DL, Deep Learning; DT, Decision Tree; Emory, Emory Risk Score; EuroSCORE, European System for Cardiac Operative Risk Evaluation; FC, Fully Connected network; GAAL, Generative Adversarial Active Learning; GBC, Gradient Boosting Classifier; GBM, Gradient Boosting Machine; GBT, Gradient Boosted Trees; GDA, Generalized Discriminant Analysis; GNB, Gaussian Naive Bayes; GRACE, Global Registry of Acute Coronary Events; Hybrid ML, Hybrid Machine Learning; iCAV-Pr, individualised Coronary Artery Visualization - Prediction; IHTSA, Index of Heart Transplant Survival After; IMPACT, Index for Mortality Prediction After Cardiac Transplantation; INTERMACS, Interagency Registry for Mechanically Assisted Circulatory Support; LGBM, Light Gradient Boosting Machine; LR, Logistic Regression; LSTM, Long Short-Term Memory network; LWL, Locally Weighted Learning; MEED, Model for Early Evaluation of Death; NB, Naive Bayes; NP, not provided; OASIS, Oxford Acute Severity of Illness Score; Papworth, Papworth Bleeding Risk Score; PRAiS2, Partial Risk Adjustment in Surgery version 2; PreOpNet, Preoperative Neural Network; PTT, partial thromboplastin time; RACHS-1, Risk Adjustment for Congenital Heart Surgery; RCRI, Revised Cardiac Risk Index; RF, Random Forest; RNN, Recurrent Neural Network; RSF, Random Survival Forest; SAPS, Simplified Acute Physiology Score; SCAD-SVM, Smoothly Clipped Absolute Deviation Support Vector Machine; SLR, Supervised Logistic Regression; SS2020, SYNTAX Score II 2020; STOP, Surgical Thoracic Operative Predictor; STS, Society of Thoracic Surgeons score; SVM, Support Vector Machine; TabBERT, Tabular BERT; TAPSE, Tricuspid Annular Plane Systolic Excursion; TextCNN, Text Convolutional Neural Network; TextLSTM, Text-based Long Short-Term Memory network; TRACK, Thoracic Risk Assessment Calculator; TRUST, Thoracic Risk Utility Scoring Tool; UAN-GVI, Uncertainty-Aware Network for Graft Versus Infection; XGBoost, eXtreme Gradient Boosting; surgVAE, Surgical Variational Autoencoder.

**References**

1. Abbasi A, Li C, Dekle M, et al. Interpretable machine learning-based predictive modeling of patient outcomes following cardiac surgery. *J Thorac Cardiovasc Surg* 2025; 169: 114–123.
2. Abdul Ghffar Y, Osman M, Shrestha S, et al. (2020). Usefulness of semisupervised machine-learning-based phenogrouping to improve risk assessment for patients undergoing transcatheter aortic valve implantation. *Am J Cardiol* 2020; 136: 122–130.
3. Agasthi P, Ashraf H, Pujari SH, et al. Artificial intelligence trumps TAVI2-SCORE and CoreValve Score in predicting 1-year mortality post-transcatheter aortic valve replacement. *Cardiovasc Revasc Med* 2021; 24: 33–41.
4. Agasthi P, Buras MR, Smith SD, et al. Machine learning helps predict long-term mortality and graft failure in patients undergoing heart transplant. *Gen Thorac Cardiovasc Surg* 2020; 68: 1369–1376.
5. Alhwiti T, Aldrugh S and Megahed FM. Predicting in-hospital mortality after transcatheter aortic valve replacement using administrative data and machine learning. *Sci Rep* 2023; 13: 10252.
6. Allou N, Allyn J, Provenchere S, et al. Clinical utility of a deep-learning mortality prediction model for cardiac surgery decision making. *J Thorac Cardiovasc Surg* 2023; 166: e567–e578.
7. Arabyarmohammadi S, Yuan C, Viswanathan VS, et al. Failing to make the grade: conventional cardiac allograft rejection grading criteria are inadequate for predicting rejection severity. *Circ Heart Fail* 2024; 17: e010950.
8. Aranda-Michel E, Sultan I, Kilic A, et al. A machine learning approach to model for end-stage liver disease score in cardiac surgery. *J Card Surg* 2022; 37: 29–38.
9. Arian F, Amini M, Mostafaei S, et al. Myocardial function prediction after coronary artery bypass grafting using MRI Radiomic features and machine learning algorithms. *J Digit Imaging* 2022; 35: 1708–1718.
10. Ayers B, Sandholm T, Gosev I, et al. Using machine learning to improve survival prediction after heart transplantation. *J Card Surg* 2021; 36: 4113–4120.
11. Bartkowiak J, Vivekanantham H, Kassar M, et al. Computed tomography anatomic predictors of outcomes in patients undergoing tricuspid transcatheter edge-to-edge repair. *J Cardiovasc Comput Tomogr* 2024; 18: 259–266.
12. Behnoush AH, Khalaji A, Rezaee M, et al. Machine learning-based prediction of 1-year mortality in hypertensive patients undergoing coronary revascularization surgery. *Clin Cardiol* 2023; 46: 269–278.
13. Benedetto U, Sinha S, Lyon M, et al. Can machine learning improve mortality prediction following cardiac surgery? *Eur J Cardiothorac Surg* 2020; 58: 1130–1136.
14. Bertsimas D, Zhuo D, Dunn J, et al. Adverse outcomes prediction for congenital heart surgery: a machine learning approach. *World J Pediatr Congenit Heart Surg* 2021; 12: 453–460.
15. Bertsimas D, Zhuo D, Levine J, et al. Benchmarking in congenital heart surgery using machine learning-derived optimal classification trees. *World J Pediatr Congenit Heart Surg* 2022; 13: 23–35.
16. Betts KS, Marathe SP, Chai K, et al. A machine learning approach to predicting 30-day mortality following paediatric cardiac surgery: findings from the Australia New Zealand Congenital Outcomes Registry for Surgery (ANZCORS). *Eur J Cardiothorac Surg* 2023; 64: ezad160.
17. Bi S, Chen S, Li J, et al. Machine learning-based prediction of in-hospital mortality for post cardiovascular surgery patients admitting to intensive care unit: a retrospective observational cohort study based on a large multi-center critical care database. *Comput Methods Programs Biomed* 2022; 226: 107115.
18. Bodenhofer U, Haslinger-Eisterer B, Minichmayer A, et al. Machine learning-based risk profile classification of patients undergoing elective heart valve surgery. *Eur J Cardiothorac Surg* 2021; 60: 1378–1385.
19. Boskovski MT, Homsy J, Nathan M, et al. De novo damaging variants, clinical phenotypes, and post-operative outcomes in congenital heart disease. *Circ Genom Precis Med* 2020; 13: e002836.
20. Cai JY, Zha ML, Song YP, et al. Predicting the development of surgery-related pressure injury using a machine learning algorithm model. *J Nurs Res* 2021: 29: e135.
21. Castela Forte J, Yeshmagambetova G, Van Der Grinten ML, et al. Comparison of machine learning models including preoperative, intraoperative, and postoperative data and mortality after cardiac surgery. *JAMA Netw Open* 2022; 5: e2237970.
22. Castela Forte J, Mungroop HE, de Geus F, et al. Ensemble machine learning prediction and variable importance analysis of 5-year mortality after cardiac valve and CABG operations. *Sci Rep* 2021; 11: 3467
23. Che J, Yang B, Xie Y, et al. A precise blood transfusion evaluation model for aortic surgery: a single-center retrospective study. *J Clin Monit Comput* 2024; 38: 691–699.
24. Chen WT, Huang HL, Ko PS, et al. A simple algorithm using ventilator parameters to predict successfully rapid weaning program in cardiac intensive care unit patients. *J Pers Med* 2022; 12: 501.
25. Chen Y, Mo Z, Chu H, et al. A model for predicting postoperative persistent acute kidney injury (AKI) in AKI after cardiac surgery patients with normal baseline renal function. *Clin Cardiol* 2024; 47: e24168.
26. Chen Z, Chen L, Yao G, et al. Novel blood cytokine-based model for predicting severe acute kidney injury and poor outcomes after cardiac surgery. *J Am Heart Assoc* 2020; 9: e018004.
27. Couto RC, Pedrosa T, Seara LM, et al. Development of a machine learning model to estimate length of stay in coronary artery bypass grafting. *Rev Saude Publica* 2024; 58: 41.
28. Cruz EO, Sakowitz S, Mallick S, et al. Application of machine learning to predict in-hospital mortality after transcatheter mitral valve repair. *Surgery* 2024; 176: 1442–1449.
29. Cruz EO, Sakowitz S, Mallick S, et al. Machine learning prediction of hospitalization costs for coronary artery bypass grafting operations. *Surgery* 2024; 176: 282–288.
30. da Cruz HF, Pfahringer B, Martensen T, et al. Using interpretability approaches to update “black-box” clinical prediction models: an external validation study in nephrology. *Artif Intell Med* 2021; 111: 101982.
31. da Cunha CBC, Lima TA, Ferraz DLM, et al. Predicting the need for blood transfusions in cardiac surgery: a comparison between machine learning algorithms and established risk scores in the Brazilian population. *Braz J Cardiovasc Surg* 2024; 39: e20230212.
32. Dai A, Zhou Z, Jiang F, et al. Incorporating intraoperative blood pressure time-series variables to assist in prediction of acute kidney injury after type A acute aortic dissection repair: an interpretable machine learning model. *Ann Med* 2023; 55: 2266458.
33. Del Gaizo J, Sherard C, Shorbaji K, et al. Prediction of coronary artery bypass graft outcomes using a single surgical note: An artificial intelligence-based prediction model study. *PLoS One* 2024; 19: e0300796.
34. Dong J, Jin Z, Li C, et al. Machine learning models with prognostic implications for predicting gastrointestinal bleeding after coronary artery bypass grafting and guiding personalized medicine: multicenter cohort study. *J Med Internet Res* 2025; 27: e68509.
35. Dong T, Oronti IB, Sinha S, et al. Enhancing cardiovascular risk prediction: development of an advanced XGBoost model with hospital-level random effects. *Bioengineering* 2024; 11: 1039.
36. Duong SQ, Shi Y, Giacone H, et al. Criteria for early pacemaker implantation in patients with postoperative heart block after congenital heart surgery. *Circ Arrhythm Electrophysiol* 2022; 15: e011145.
37. El Ouahidi A, El Ouahidi Y, Nicol PP, et al. Machine learning for pacemaker implantation prediction after TAVI using multimodal imaging data. *Sci Rep* 2024; 14: 25008.
38. Elmahrouk A, Daoulah A, Panduranga P, et al. Leveraging machine learning to enhance postoperative risk assessment in coronary artery bypass grafting patients with unprotected left main disease: a retrospective cohort study. *Int J Surg* 2024; 110: 7142.
39. Evertz R, Lange T, Backhaus SJ, et al. Artificial intelligence enabled fully automated CMR function quantification for optimized risk stratification in patients undergoing transcatheter aortic valve replacement. *J Interv Cardiol* 2022; 2022: 1368878.
40. Fan R, Qin W, Zhang H, et al. Machine learning in the prediction of cardiac surgery associated acute kidney injury with early postoperative biomarkers. *Front Surg* 2023; 10: 1048431.
41. Fan Y, Dong J, Wu Y, et al. Development of machine learning models for mortality risk prediction after cardiac surgery. *Cardiovasc Diagn Ther* 2022; 12: 12–23.
42. Fernandes MPB, Armengol de la Hoz M, Rangasamy V, et al. Machine learning models with preoperative risk factors and intraoperative hypotension parameters predict mortality after cardiac surgery. *J Cardiothorac Vasc Anesth* 2021; 35: 857–865.
43. Fliegenschmidt J, Hulde N, Preising MG, et al. Leveraging artificial intelligence for the management of postoperative delirium following cardiac surgery. *Eur J Anaesthesiol Intensive Care* 2022; 2: e0010.
44. Fragasso T, Raggi V, Passaro D, et al. Predicting acute kidney injury with an artificial intelligence-driven model in a pediatric cardiac intensive care unit. *J Anesth Analg Crit Care* 2023; 3: 37.
45. Gao P, Zhang Y, Jin Y, et al. Development and validation of a nomogram for predicting heparin resistance in neonates and young infants undergoing cardiac surgery: a retrospective study. *Anesth Analg* 2023; 138: 1233.
46. Giangreco NP, Lebreton G, Restaino S, et al. Plasma kallikrein predicts primary graft dysfunction after heart transplant. *J Heart Lung Transplant* 2021; 40: 1199–1211.
47. Gomes B, Pilz M, Reich C, et al. Machine learning-based risk prediction of intrahospital clinical outcomes in patients undergoing TAVI. *Clin Res Cardiol* 2021; 110: 343–356.
48. Griffeth EM, Stephens EH, Dearani JA, et al. Impact of heart failure on reoperation in adult congenital heart disease: an innovative machine learning model. *J Thorac Cardiovasc Surg* 2024; 167: 2215–2225.
49. Habertheuer A, Geirsson A, Gleason T, et al. STratification risk analysis in OPerative management (STOP score) for drug-induced endocarditis. *J Card Surg* 2021; 36: 2442–2451.
50. Halloran PF, Madill-Thomsen K, Aliabadi-Zuckermann AZ, et al. Redefining the molecular rejection states in 3230 heart transplant biopsies: relationships to parenchymal injury and graft survival. *Am J Transplant* 2024; 24: 1414–1426.
51. Haregu F, Dixon RJ, McCulloch M, et al. Machine learning for predicting waitlist mortality in pediatric heart transplantation. *Pediatr Transplant* 2025; 29: e70095.
52. Hausleiter J, Lachmann M, Stolz L, et al. Artificial intelligence-derived risk score for mortality in secondary mitral regurgitation treated by transcatheter edge-to-edge repair: the EuroSMR risk score. *Eur Heart J* 2024; 45: 922–936.
53. Hui V, Litton E, Edibam C, et al. Using machine learning to predict bleeding after cardiac surgery. *Eur J Cardiothorac Surg* 2023; 64: ezad297.
54. Hur C, Wi J and Kim Y. Facilitating the development of deep learning models with visual analytics for electronic health records. *Int J Environ Res Public Health* 2020; 17: 1–14.
55. Jia T, Xu K, Bai Y, et al. Machine-learning predictions for acute kidney injuries after coronary artery bypass grafting: a real-life muticenter retrospective cohort study. *BMC Med Inform Decis Mak* 2023; 23: 270.
56. Jiang J, Liu X, Cheng Z, et al. Interpretable machine learning models for early prediction of acute kidney injury after cardiac surgery. *BMC Nephrol* 2023; 24: 326.
57. Jiang X, Peng W, Xu J, et al. Development and validation of machine learning models for predicting extubation failure in patients undergoing cardiac surgery: a retrospective study. *Sci Rep* 2025; 15: 8506.
58. Kachroo P, Guo A, MacGregor RM, et al. Association of STS database variables with repair durability in ischemic mitral regurgitation using machine learning. *J Card Surg* 2022; 37: 76–83.
59. Kageyama S, Ninomiya K, Jonik S, et al. Systematic screening by a heart team and a machine learning approach contribute to unraveling novel risk factors in revascularization candidates with complex coronary artery disease. *Pol Arch Intern Med* 2024; 134: 16747.
60. Kainuma A, Ning Y, Kurlansky PA, et al. Predictors of 1-year outcome after cardiac re-transplantation: machine learning analysis. *Clin Transplant* 2022; 36: e14761.
61. Kalisnik JM, Bauer A, Vogt FA, et al. Artificial intelligence-based early detection of acute kidney injury after cardiac surgery. *Eur J Cardiothorac Surg* 2022; 62: ezac289.
62. Kampaktsis PN, Siouras A, Doulamis IP, et al. Machine learning-based prediction of mortality after heart transplantation in adults with congenital heart disease: a UNOS database analysis. *Clin Transplant* 2023; 37: e14761.
63. Kampaktsis PN, Tzani A, Doulamis IP, et al. State-of-the-art machine learning algorithms for the prediction of outcomes after contemporary heart transplantation: results from the UNOS database. *Clin Transplant* 2021; 35: e14388.
64. Karri R, Kawai A, Thong YJ, et al. Machine learning outperforms existing clinical scoring tools in the prediction of postoperative atrial fibrillation during intensive care unit admission after cardiac surgery. *Heart Lung Circ* 2021; 30: 1929–1937.
65. Kilic A, Goyal A, Miller JK, et al. Performance of a machine learning algorithm in predicting outcomes of aortic valve replacement. *Ann Thorac Surg* 2021; 111: 503–510.
66. Kilic A, Habib RH, Miller JK, et al. Supplementing existing societal risk models for surgical aortic valve replacement with machine learning for improved prediction. *J Am Heart Assoc* 2021; 10: e019697.
67. Kim J and Jang I. Predictors of bleeding event among elderly patients with mechanical valve replacement using random forest model: a retrospective study. *Medicine* 2021; 100: E25875.
68. Kim RB, Alge OP, Liu G, et al. Prediction of postoperative cardiac events in multiple surgical cohorts using a multimodal and integrative decision support system. *Sci Rep* 2022; 12: 11347.
69. Kirchner J, Gerçek M, Gesch J, et al. Artificial intelligence-analyzed computed tomography in patients undergoing transcatheter tricuspid valve repair. *Int J Cardiol* 2024; 411: 132233.
70. Kobayashi Y, Peng YC, Yu E, et al. Prediction of lactate concentrations after cardiac surgery using machine learning and deep learning approaches. *Front Med* 2023; 10: 1165912.
71. Kurmanaliyev A, Sutiene K, Braukylienė R, et al. An integrative machine learning model for predicting early safety outcomes in patients undergoing transcatheter aortic valve implantation. *Medicina* 2025; 61: 374.
72. Kwak S, Everett RJ, Treibel TA, et al. Markers of myocardial damage predict mortality in patients with aortic stenosis. *J Am Coll Cardiol* 2021; 78: 545–558.
73. Lachmann M, Fortmeier V, Stolz L, et al. Deep learning-enabled assessment of right ventricular function improves prognostication after transcatheter edge-to-edge repair for mitral regurgitation. *Circ Cardiovasc Imaging* 2025; 18: 17005.
74. Lachmann M, Rippen E, Schuster T, et al. Artificial intelligence-enabled phenotyping of patients with severe aortic stenosis: on the recovery of extra-aortic valve cardiac damage after transcatheter aortic valve replacement. *Open Heart* 2022; 9: e002068.
75. Li L, Hu Y, Yang Z, et al. Exploring the assessment of post-cardiac valve surgery pulmonary complication risks through the integration of wearable continuous physiological and clinical data. *BMC Med Inform Decis Mak* 2025; 25: 47.
76. Li Q, Li J, Chen J, et al. A machine learning-based prediction model for postoperative delirium in cardiac valve surgery using electronic health records. *BMC Cardiovasc Disord* 2024; 24: 56.
77. Li Q, Lv H, Chen Y, et al. Development and validation of a machine learning predictive model for cardiac surgery-associated acute kidney injury. *J Clin Med* 2023; 12: 1166.
78. Li Q, Lv H, Chen Y, et al. Development and validation of a machine learning predictive model for perioperative myocardial injury in cardiac surgery with cardiopulmonary bypass. *J Cardiothorac Surg* 2024; 19: 384.
79. Li R, Shen L, Ma W, et al. Use of machine learning models to predict in-hospital mortality in patients with acute coronary syndrome. *Clin Cardiol* 2023: 46: 184–194.
80. Li T, Yang Y, Huang J, et al. Machine learning to predict post-operative acute kidney injury stage 3 after heart transplantation. *BMC Cardiovasc Disord* 2022; 22: 288.
81. Li Y, Xu J, Wang Y, et al. A novel machine learning algorithm, Bayesian networks model, to predict the high-risk patients with cardiac surgery-associated acute kidney injury. *Clin Cardiol* 2020; 43: 752–761.
82. Li Z, Fan J, Fan J, et al. Risk factors and predictive models for post-operative moderate-to-severe mitral regurgitation following transcatheter aortic valve replacement: a machine learning approach. *BMC Cardiovasc Disord* 2025; 25: 361.
83. Liou L, Mostofsky E, Lehman L, et al. Survival machine learning methods for mortality prediction after heart transplantation in the contemporary era. *PLoS One* 2025; 20: e0313600.
84. Lisboa PJG, Jayabalan M, Ortega-Martorell S, et al. Enhanced survival prediction using explainable artificial intelligence in heart transplantation. *Sci Rep* 2022; 12: 19525.
85. Liu K, Zhang X, Chen W, et al. Development and validation of a personalized model with transfer learning for acute kidney injury risk estimation using electronic health records. *JAMA Netw Open* 2022; 5: e2219776.
86. Liu S, Su L, Jiang H, et al. Full connected layer model with self-attention to hourly predict heparin dosage for perioperative cardiac surgery patients. *Comput Biol Med* 2024; 182: 109085.
87. Lu Y, Chen Q, Zhang H, et al. Machine learning models of postoperative atrial fibrillation prediction after cardiac surgery. *J Cardiothorac Vasc Anesth* 2023; 37: 360–366.
88. Luo L, Huang SQ, Liu C, et al. Machine learning–based risk model for predicting early mortality after surgery for infective endocarditis. *J Am Heart Assoc* 2022; 11: e025433.
89. Luo XQ, Kang YX, Duan SB, et al. Machine learning–based prediction of acute kidney injury following pediatric cardiac surgery: model development and validation study. *J Med Internet Res* 2023; 25: e41142.
90. Ma W, Li H, Dong L, et al. Warfarin maintenance dose prediction for Chinese after heart valve replacement by a feedforward neural network with equal stratified sampling. *Sci Rep* 2021; 11: 13778.
91. Mao Y, Liu Y, Zhai M, et al. Clinical value of aortic arch morphology in transfemoral TAVR: artificial intelligence evaluation. *Int J Surg* 2025; 111: 2338–2347.
92. Marmagkiolis K, Monlezun DJ, Caballero J, et al. Prevalence, mortality, cost, and disparities in transcatheter mitral valve repair and replacement in cancer patients: artificial intelligence and propensity score national 5-year analysis of 7495 procedures. *Int J Cardiol* 2024; 408: 132091
93. Mathis MR, Engoren MC, Williams AM, et al. Prediction of postoperative deterioration in cardiac surgery patients using electronic health record and physiologic waveform data. *Anesthesiology* 2022; 137: 586–601.
94. Mohammadyari P, Vieceli Dalla Sega F, Fortini F, et al. Deep-learning survival analysis for patients with calcific aortic valve disease undergoing valve replacement. *Sci Rep* 2024; 14: 10902.
95. Molina RS, Molina-Rodríguez MA, Rincón FM, et al. Cardiac operative risk in Latin America: a comparison of machine learning models vs EuroSCORE-II. *Ann Thorac Surg* 2022; 113: 92–99.
96. Mori M, Durant TJS, Huang C, et al. Toward dynamic risk prediction of outcomes after coronary artery bypass graft: improving risk prediction with intraoperative events using gradient boosting. *Circ Cardiovasc Qual Outcomes* 2021; 14: E007363.
97. Mustafa A, Wei C, Grovu R, et al. Using novel machine learning tools to predict optimal discharge following transcatheter aortic valve replacement. *Arch Cardiovasc Dis* 2025; 118: 26–34.
98. Nagy M, Onder AM, Rosen D, et al. Predicting pediatric cardiac surgery-associated acute kidney injury using machine learning. *Pediatr Nephrol* 2024; 39: 1263–1270.
99. Namasivayam M, Myers PD, Guttag JV, et al. Predicting outcomes in patients with aortic stenosis using machine learning: the Aortic Stenosis Risk (ASteRisk) score. *Open Heart* 2022; 9: e001990.
100. Narayan P, Swamy AK, Ghorai PA, et al. Effect of preoperative statins on respiratory complications after coronary artery bypass grafting. *J Card Surg* 2022; 37: 4639–4645.
101. Nistal-Nuño, B. Machine learning applied to a Cardiac Surgery Recovery Unit and to a Coronary Care Unit for mortality prediction. *J Clin Monit Comput* 2022; 36: 751–763.
102. Nowakowska K, Sakellarios A, Kaźmierski J, et al. AI-enhanced predictive modeling for identifying depression and delirium in cardiovascular patients scheduled for cardiac surgery. *Diagnostics* 2024; 14: 67.
103. Okuno T, Overtchouk P, Asami M, et al. Deep learning-based prediction of early cerebrovascular events after transcatheter aortic valve replacement. *Sci Rep* 2021; 11: 18754.
104. Orfanoudaki A, Giannoutsou A, Hashim S, et al. Machine learning models for mitral valve replacement: a comparative analysis with the Society of Thoracic Surgeons risk score. *J Card Surg* 2022; 37: 18–28.
105. Ouyang D, Theurer J, Stein NR, et al. Electrocardiographic deep learning for predicting post-procedural mortality: a model development and validation study. *Lancet Digit Health* 2024; 6: e70–e78.
106. Pan T, Zhang H, Wang C, et al. Development, validation, and clinical evaluation of a machine-learning based model for diagnosing early infection after cardiovascular surgery (DEICS): a multi-center cohort study. *Int J Surg* 2025; 111: 2862–2871.
107. Pei Y, Ma Y, Xiang Y, et al. Stress hyperglycemia ratio and machine learning model for prediction of all-cause mortality in patients undergoing cardiac surgery. *Cardiovasc Diabetol* 2025; 24: 77.
108. Pekař M, Jiravský O, Novák J, et al. Sarcopenia and adipose tissue evaluation by artificial intelligence predicts the overall survival after TAVI. *Sci Rep* 2024; 14: 8842.
109. Penny-Dimri JC, Bergmeir C, Reid CM, et al. Machine learning algorithms for predicting and risk profiling of cardiac surgery-associated acute kidney injury. *Semin Thorac Cardiovasc Surg* 2021; 33: 735–745.
110. Penny-Dimri JC, Bergmeir C, Reid CM, et al. Paying attention to cardiac surgical risk: an interpretable machine learning approach using an uncertainty-aware attentive neural network. *PLoS One* 2023; 18: e0289930.
111. Perduca V, Bouaziz O, Zannis K, et al. Can machine learning provide preoperative predictions of biological hemostasis after extracorporeal circulation for cardiac surgery? *J Thorac Cardiovasc Surg* 2024; 168: 1120–1129.
112. Petrosyan Y, Mesana TG and Sun LY. Prediction of acute kidney injury risk after cardiac surgery: using a hybrid machine learning algorithm. *BMC Med Inform Decis Mak* 2022; 22: 137.
113. Peyster EG, Janowczyk A, Swamidoss A, et al. Computational analysis of routine biopsies improves diagnosis and prediction of cardiac allograft vasculopathy. *Circulation* 2022; 145: 1563–1577.
114. Raghu VK, Moonsamy P, Sundt TM, et al. Deep learning to predict mortality after cardiothoracic surgery using preoperative chest radiographs. Ann Thorac Surg 2023; 115: 257–264.
115. Rank N, Pfahringer B, Kempfert J, et al. Deep-learning-based real-time prediction of acute kidney injury outperforms human predictive performance. *NPJ Digit Med* 2020; 3: 139.
116. Rogers MP, Janjua H, Fishberger G, et al. A machine learning approach to high-risk cardiac surgery risk scoring. *J Card Surg* 2022; 37: 4612–4620.
117. Ryan CT, Zeng Z, Chatterjee S, et al. Machine learning for dynamic and early prediction of acute kidney injury after cardiac surgery. *J Thorac Cardiovasc Surg* 2023; 166: e551–e564.
118. Santos R, Ribeiro B, Sousa I, et al. Predicting post-discharge complications in cardiothoracic surgery: a clinical decision support system to optimize remote patient monitoring resources. *Int J Med Inform* 2024; 182: 105307.
119. Sarris GE, Zhuo D, Mingardi L, et al. Congenital heart surgery machine learning-derived in-depth benchmarking tool. *Ann Thorac Surg* 2024; 118: 199–206.
120. Schuurmans J, Rellum SR, Schenk J, et al. Effect of a machine learning-derived early warning tool with treatment protocol on hypotension during cardiac surgery and ICU stay: the Hypotension Prediction 2 (HYPE-2) randomized clinical trial. *Crit Care Med* 2025; 53: e328–e340.
121. Sen E and Seckiner SU. The use of artificial neural networks to determine in-hospital mortality after coronary artery bypass surgery. *J Cardiothorac Vasc Anesth* 2021; 35: 2432–2437.
122. Shen J, Xue B, Kannampallil T, et al. A novel generative multi-task representation learning approach for predicting postoperative complications in cardiac surgery patients. *J Am Med Inform Assoc* 2025; 32: 459–469.
123. Sherman E, Alejo D, Wood-Doughty Z, et al. Leveraging machine learning to predict 30-day hospital readmission after cardiac surgery. *Ann Thorac Surg* 2022; 114: 2173–2179.
124. Sinha S, Dong T, Dimagli A, et al. A machine learning algorithm-based risk prediction score for in-hospital/30-day mortality after adult cardiac surgery. *Eur J Cardiothorac Surg* 2024; 66: ezae368.
125. Sinha S, Dong T, Dimagli A, et al. Comparison of machine learning techniques in prediction of mortality following cardiac surgery: analysis of over 220 000 patients from a large national database. *Eur J Cardiothorac Surg* 2023; 63: ezad183.
126. Song W, Frakes D and Dasi LP. Active machine learning for pre-procedural prediction of time-varying boundary condition after fontan procedure using generative adversarial networks. *Ann Biomed Eng* 2025; 53: 217–229.
127. Song Y, Zhai W, Ma S, et al. Machine learning-based prediction of off-pump coronary artery bypass grafting-associated acute kidney injury. *J Thorac Dis* 2024; 16: 4535–4542.
128. Squiccimarro E, Lorusso R, Consiglio A, et al. Impact of inflammation after cardiac surgery on 30-day mortality and machine learning risk prediction. *J Cardiothorac Vasc Anesth* 2025; 39: 683–691.
129. Stošić M, Perić V, Milić D, et al. Analyzing key predictors of postoperative delirium following coronary artery bypass grafting and aortic valve replacement: a machine learning perspective. *Medicina* 2025; 61: 883.
130. Sudri K, Motro-Feingold I, Ramon-Gonen R, et al. Enhancing coronary revascularization decisions: the promising role of large language models as a decision-support tool for multidisciplinary heart team. *Circ Cardiovasc Interv* 2024; 17: e014201.
131. Sughimoto K, Levman J, Baig F, et al. Machine learning predicts blood lactate levels in children after cardiac surgery in paediatric ICU. *Cardiol Young* 2023; 33: 388–395.
132. Sun Z, Cui Z, Xie Y, et al. Evaluation of the factors influencing blood transfusion during minimally invasive direct coronary artery bypass surgery. *Cardiology* 2025; 150: 98–110.
133. Tadege M, Tegegne AS and Dessie ZG. Cardiac patients’ surgery outcome and associated factors in Ethiopia: application of machine learning. *BMC Pediatr* 2024; 24: 395.
134. Tadege M, Tegegne AS and Dessie ZG. Post-surgery survival and associated factors for cardiac patients in Ethiopia: applications of machine learning, semi-parametric and parametric modelling. *BMC Med Inform Decis Mak* 2024; 24: 91.
135. Tang B, He K, Liu S, et al. Preoperative ECG-assisted feature engineering enhances prediction of new-onset atrial fibrillation after cardiac surgery. *Comput Methods Programs Biomed* 2025; 264: 108696.
136. Tang X, Wang T, Shi H, et al. Artificial intelligence and big data technologies in the construction of surgical risk prediction model for patients with coronary artery bypass grafting. *Comput Intell Neurosci* 2023; 2023: 9575553.
137. Theis M, Block W, Luetkens JA, et al. Direct deep learning-based survival prediction from pre-interventional CT prior to transcatheter aortic valve replacement. *Eur J Radiol* 2023; 168: 111150.
138. Thongprayoon C, Pattharanitima P, Kattah AG, et al. Explainable preoperative automated machine learning prediction model for cardiac surgery-associated acute kidney injury. *J Clin Med* 2022; 11: 6264.
139. Tong C, Du X, Chen Y, et al. Machine learning prediction model of major adverse outcomes after pediatric congenital heart surgery: a retrospective cohort study. *Int J Surg* 2024; 110: 2207–2216.
140. Topkara VK, Elias P, Jain R, et al. Machine learning-based prediction of myocardial recovery in patients with left ventricular assist device support. *Circ Heart Fail* 2022; 15: 20–27.
141. Triana AJ, Vyas R, Shah AS, et al. Predicting length of stay of coronary artery bypass grafting patients using machine learning. *J Surg Res* 2021; 264: 68–75.
142. Truong VT, Beyerbach D, Mazur W, et al. Machine learning method for predicting pacemaker implantation following transcatheter aortic valve replacement. *Pacing Clin Electrophysiol* 2021; 44: 334–340.
143. Tseng PY, Chen YT, Wang CH, et al. Prediction of the development of acute kidney injury following cardiac surgery by machine learning. *Crit Care* 2020; 24: 478.
144. Tsushima T, Al-Kindi S, Nadeem F, et al. Machine learning algorithms for prediction of permanent pacemaker implantation after transcatheter aortic valve replacement. *Circ Arrhythm Electrophysiol* 2021; 14: E008941.
145. van Erck D, Moeskops P, Schoufour JD, et al. Low muscle quality on a procedural computed tomography scan assessed with deep learning as a practical useful predictor of mortality in patients with severe aortic valve stenosis. *Clin Nutr ESPEN* 2024; 63: 142–147.
146. Vasileios C, Giorgos F, Antonios M, et al. AI-based prediction of left bundle branch block risk post-TAVI using pre-implantation clinical parameters. *Future Cardiol* 2025; 21: 489–494.
147. Wang J, Wang Y, Duan S, et al. Multimodal data-driven prognostic model for predicting long-term prognosis in patients with ischemic cardiomyopathy and heart failure with preserved ejection fraction after coronary artery bypass grafting: a multicenter cohort study. *J Am Heart Assoc* 2024; 13: 36970.
148. Wang J, Zhu J, Li H, et al. Multimodal visualization and explainable machine learning-driven markers enable early identification and prognosis prediction for symptomatic aortic stenosis and heart failure with preserved ejection fraction after transcatheter aortic valve replacement: multicenter cohort study. *J Med Internet Res* 2025; 27: e70587.
149. Wang R, Pan D, Sun X, et al. Two birds with one stone: pre-TAVI coronary CT angiography combined with FFR helps screen for coronary stenosis. *BMC Med Imaging* 2025; 25: 192.
150. Wang Z, Zhe S, Zimmerman J, et al. Development and validation of a machine learning method to predict intraoperative red blood cell transfusions in cardiothoracic surgery. *Sci Rep* 2022; 12: 1355.
151. Wayda B, Weng Y, Zhang S, et al. Prediction of donor heart acceptance for transplant and its clinical implications: results from the Donor Heart Study. *Circ Heart Fail* 2024; 17: e011360.
152. Wisotzkey BL, Jaeger B, Asante-Korang A, et al. Risk factors for 1-year allograft loss in pediatric heart transplant patients using machine learning: an analysis of the pediatric heart transplant society database. *Pediatr Transplant* 2023; 27: e14612.
153. Woillard JB, Labriffe M, Debord J, et al. Mycophenolic acid exposure prediction using machine learning. *Clin Pharmacol Ther* 2021; 110: 370–379.
154. Xue L, He S, Singla RK, et al. Machine learning guided prediction of warfarin blood levels for personalized medicine based on clinical longitudinal data from cardiac surgery patients: a prospective observational study. *Int J Surg* 2024; 110: 6528–6540.
155. Yang C, Zheng P, Li L, et al. Machine learning-based model development for predicting risk factors of prolonged intra-aortic balloon pump therapy in patients with coronary artery bypass grafting. *J Cardiothorac Surg* 2024; 19: 383.
156. Yang C, Zheng P, Zhang Q, et al. Machine learning model for risk prediction of prolonged intensive care unit in patients receiving intra-aortic balloon pump therapy during coronary artery bypass graft surgery. *J Cardiovasc Transl Res* 2025; 18: 341–353.
157. Yang HL, Jung CW, Yang SM, et al. Development and validation of an arterial pressure-based cardiac output algorithm using a convolutional neural network: retrospective study based on prospective registry data. *JMIR Med Inform* 2021; 9: e24762.
158. Yang T, Yang H, Liu Y, et al. Postoperative delirium prediction after cardiac surgery using machine learning models. *Comput Biol Med* 2024; 169: 107818.
159. Yang Y, Yan Y, Zhou Z, et al. Accurate prediction of bleeding risk after coronary artery bypass grafting with dual antiplatelet therapy: a machine learning model vs. the PRECISE-DAPT score. *Int J Cardiol* 2025; 421: 132925.
160. Yerukala Sathipati S, Carter T, Soodi D, et al. MicroRNA signature predicts post operative atrial fibrillation after coronary artery bypass grafting. *Sci Rep* 2025; 15: 18658.
161. Yu J, Taskén AA, Berg EAR, et al. Continuous monitoring of left ventricular function in postoperative intensive care patients using artificial intelligence and transesophageal echocardiography. *Intensive Care Med Exp* 2024; 12: 54.
162. Zea-Vera R, Ryan CT, Havelka J, et al. Machine learning to predict outcomes and cost by phase of care after coronary artery bypass grafting. *Ann Thorac Surg* 2022; 114: 711–719.
163. Zea-Vera R, Ryan CT, Navarro SM, et al. Development of a machine learning model to predict outcomes and cost after cardiac surgery. *Ann Thorac Surg* 2023; 115: 1533–1542.
164. Zeng J, Zhang D, Lin S, et al. Comparative analysis of machine learning vs. traditional modeling approaches for predicting in-hospital mortality after cardiac surgery: temporal and spatial external validation based on a nationwide cardiac surgery registry. *Eur Heart J Qual Care Clin Outcomes* 2024; 10: 121–131.
165. Zeng X, Hu Y, Shu L, et al. Explainable machine-learning predictions for complications after pediatric congenital heart surgery. *Sci Rep* 2021; 11: 17244.
166. Zeng X, Shi S, Sun Y, et al. A time-aware attention model for prediction of acute kidney injury after pediatric cardiac surgery. *J Am Med Inform Assoc* 2023; 30: 94–102.
167. Zeng Z, Tian X, Li L, et al. An interpretable machine learning model to predict off-pump coronary artery bypass grafting-associated acute kidney injury. *Adv Clin Exp Med* 2024; 33: 473–481.
168. Zhang H, Qian D, Zhang X, et al. Tree-based ensemble machine learning models in the prediction of acute respiratory distress syndrome following cardiac surgery: a multicenter cohort study. *J Transl Med* 2024; 22: 772.
169. Zhang L, Zhou X and Cao J. Establishment and validation of a heart failure risk prediction model for elderly patients after coronary rotational atherectomy based on machine learning. *PeerJ* 2024; 12: e16867.
170. Zhang Q, Zheng P, Hong Z, et al. Machine learning in risk prediction of continuous renal replacement therapy after coronary artery bypass grafting surgery in patients. *Clin Exp Nephrol* 2024; 28: 811–821.
171. Zhang Y, Li L, Li Y, et al. Machine learning model-based risk prediction of severe complications after off-pump coronary artery bypass grafting. *Adv Clin Exp Med* 2023; 32: 185–194.
172. Zhao X, Li J, Xie X, et al. Online interpretable dynamic prediction models for postoperative delirium after cardiac surgery under cardiopulmonary bypass developed based on machine learning algorithms: a retrospective cohort study. *J Psychosom Res* 2024; 176: 111553.
173. Zhong Z, Yuan X, Liu S, et al. Machine learning prediction models for prognosis of critically ill patients after open-heart surgery. *Sci Rep* 2021; 11: 3384.
174. Zhou MY, Shi YB, Bai SJ, et al. Functional MRI-based machine learning strategy for prediction of postoperative delirium in cardiac surgery patients: a secondary analysis of a prospective observational study. *J Clin Anesth* 2025; 102: 111771.
175. Zhou Y, Chen S, Rao Z, et al. Prediction of 1-year mortality after heart transplantation using machine learning approaches: a single-center study from China. *Int J Cardiol* 2021; 339: 21–27.
176. Zhou Z, Jian B, Chen X, et al. Heterogeneous treatment effects of coronary artery bypass grafting in ischemic cardiomyopathy: a machine learning causal forest analysis. *J Thorac Cardiovasc Surg* 2024; 168: 1462–1471.
177. Zsarnoczay E, Varga-Szemes A, Schoepf UJ, et al. Predicting mortality after transcatheter aortic valve replacement using AI-based fully automated left atrioventricular coupling index. *J Cardiovasc Comput Tomogr* 2025; 19: 201–207.
178. Zweck E, Spieker M, Horn P, et al. Machine learning identifies clinical parameters to predict mortality in patients undergoing transcatheter mitral valve repair. *JACC Cardiovasc Interv* 2021; 14: 2027–2036.
